# The classical NLRP3 inflammasome controls FADD unconventional secretion through microvesicle shedding

**DOI:** 10.1038/s41419-019-1412-9

**Published:** 2019-02-25

**Authors:** Sara Mouasni, Virginie Gonzalez, Alain Schmitt, Evangeline Bennana, François Guillonneau, Sylvie Mistou, Jérôme Avouac, Hang Korng Ea, Valérie Devauchelle, Jacques-Eric Gottenberg, Gilles Chiocchia, Léa Tourneur

**Affiliations:** 1 Department of Infection, Immunity and Inflammation, Cochin Institute, 75014 Paris, France; 2 Inserm, U1016 Paris, France; 3 Cnrs, UMR8104 Paris, France; 4 Paris Descartes University, Sorbonne Paris Cité, Paris, France; 5 Cochin Imaging: Electron Microscopy Facility, 75014 Paris, France; 6 3P5 Proteomics Facility, 75014 Paris, France; 7 Rheumatology Department, AP-HP, Cochin Hospital, 75014 Paris, France; 8 Rheumatology Department, Viggo-Petersen Center, AP-HP, Lariboisière Hospital, 75010 Paris, France; 9 University School of Medicine, University Paris-Cité Paris-Diderot, Paris, France; 10 INSERM UMR-S U1132, Paris, France; 11 Rheumatology Department, CHU la Cavale Blanche, 29200 Brest, France; 12 Rheumatology Department, Strasbourg University Hospitals, 67000 Strasbourg, France; 13 Inserm U1173, University of Versailles-Saint-Quentin, Saint-Quentin-En-Yvelines, France; 14 UFR des Sciences de la Santé, Simone Veil, 78180 Montigny-Le-Bretonneux, France

## Abstract

Fas-associated death domain (FADD) is a key adaptor molecule involved in numerous physiological processes including cell death, proliferation, innate immunity and inflammation. Therefore, changes in FADD expression have dramatic cellular consequences. In mice and humans, FADD regulation can occur through protein secretion. However, the molecular mechanisms accounting for human FADD secretion were still unknown. Here we report that canonical, non-canonical, but not alternative, NLRP3 inflammasome activation in human monocytes/macrophages induced FADD secretion. NLRP3 inflammasome activation by the bacterial toxin nigericin led to the proinflammatory interleukin-1β (IL-1β) release and to the induction of cell death by pyroptosis. However, we showed that FADD secretion could occur in absence of increased IL-1β release and pyroptosis and, reciprocally, that IL-1β release and pyroptosis could occur in absence of FADD secretion. Especially, FADD, but not IL-1β, secretion following NLRP3 inflammasome activation required extracellular glucose. Thus, FADD secretion was an active process distinct from unspecific release of proteins during pyroptosis. This FADD secretion process required K^+^ efflux, NLRP3 sensor, ASC adaptor and CASPASE-1 molecule. Moreover, we identified FADD as a leaderless protein unconventionally secreted through microvesicle shedding, but not exosome release. Finally, we established human soluble FADD as a new marker of joint inflammation in gout and rheumatoid arthritis, two rheumatic diseases involving the NLRP3 inflammasome. Whether soluble FADD could be an actor in these diseases remains to be determined. Nevertheless, our results advance our understanding of the mechanisms contributing to the regulation of the FADD protein expression in human cells.

## Introduction

The Fas-Associated Death Domain (FADD) protein is the key adaptor molecule for the death receptors of the tumor necrosis factor receptor (TNF-R) superfamily. Besides being an essential component of several death signaling pathways, FADD is also involved in numerous physio-pathological processes including cancer development, innate immunity and inflammation^1,2^. Thus, FADD expression modulation have dramatic cellular consequences^3–9^.

Since many years, FADD has been described as a regulator of the inflammatory processes^1,3,10,11^. FADD contributes to the NLRP3/NALP3/cryopyrin inflammasome activation^12,13^. The NLRP3 inflammasome is a cytosolic multiprotein complex assembling in innate immune cells, such as monocytes/macrophages in response to stress or danger signals^14,15^. It consists mainly of the intracellular sensor NLRP3, the adaptor ASC (apoptosis-associated speck-like protein containing a caspase-recruitment domain) and the pro-CASPASE-1^16^. Inflammasome assembly leads to the activation of CASPASE-1-mediated cleavage and unconventional secretion of proinflammatory cytokines interleukin-1β (IL-1β) and IL-18^17^, as well as the initiation of pyroptosis, an inflammatory cell death^18^. Full NLRP3 inflammasome activation requires both priming and activation steps. Toll-like receptor (TLR) agonists such as lipopolysaccharide (LPS) induce a dispensable transcriptional priming, whereas numerous infectious and stress-associated signals including bacterial toxin nigericin, ATP, and crystals, trigger its activation^19^. Besides this canonical pathway, a non-canonical pathway induced by bacterial enteropathogens and requiring CASPASE-11 in mice or CASPASEs-4/5 in humans exists^20,21^. Upon activation by intracellular LPS from phagocytosed bacteria, inflammatory CASPASE-4 cleaves the pore-forming protein GSDMD (Gasdermin D) and activates the NLRP3 inflammasome. Formation of GSDMD pores at the membrane leads to cellular content release and pyroptosis^22^. FADD mediates both priming and activation of the canonical and non-canonical NLRP3 inflammasome in mice^12^. Decreased cytosolic potassium is the only common mechanism identified for CASPASE-1 activation by stimuli leading to NLRP3 inflammasomme activation^23,24^. However, LPS triggers an alternative NLRP3 inflammasome occurring in absence of K^+^ efflux exclusively in human monocytes. This alternative inflammasome involves a RIPK1 (receptor-interacting serine/threonine-protein kinase)-FADD-CASPASE-8 signaling occurring upstream of the classical NLRP3-ASC-CASPASE-1 signaling^13,25^. Thus, FADD participates to the canonical, non-canonical, and alternative NLRP3 inflammasome signaling leading to IL-1β secretion.

IL-1β is secreted by unconventional protein secretion (UPS), an endoplasmic reticulum (ER)/Golgi-independent mechanism^26,27^. Different mechanisms account for UPS of IL-1β in macrophages^28^ including secretory lysosomes, microvesicle shedding, exosome release, secretory autophagy, passive release during cell death, plasma membrane translocation via transporter or pore formation^29,30^. During microvesicle shedding, both pro-IL-1β and pro-CASPASE-1 are packaged into microvesicles shed from the plasma membrane^31^. CASPASE-1 then cleaves and activates IL-1β, which is delivered to extracellular space upon microvesicle burst. Alternatively, pro-IL-1β and pro-CASPASE-1 can be packaged into multivesicular bodies and released by cells within smaller vesicles called exosomes^32^.

FADD has been detected both in the nucleus and the cytoplasm^33^. Additionally, we found an unexpected localization of FADD into the extracellular compartment, demonstrating that FADD protein can be secreted^1^. In humans, FADD secretion correlated with cancer development and aggressiveness, emphasizing FADD importance in pathological processes^34^. In mice, FADD secretion can occur through microvesicle shedding and involves adenosine receptors^35^. However, the mechanism accounting for FADD secretion by human cells was still unknown. FADD participates to the canonical, non-canonical, and alternative NLRP3 inflammasome signaling^12,13^. Proteins belonging to the NLRP3 inflammasome complex like ASC^36^, CASPASE-1, and NLRP3^37^ can be co-secreted with IL-1β following inflammasome activation. Here, we demonstrate that human monocytes/macrophages unconventionally secrete FADD protein through microvesicle shedding under the control of the classical NLRP3 inflammasome. Moreover, our results establish human soluble FADD as a marker of joint inflammation during rheumatoid arthritis (RA) and gout attack, two inflammatory rheumatic diseases involving the NLRP3 inflammasome^38–40^.

## Results

### Activation of the classical NLRP3 inflammasome induces FADD protein secretion from human monocytes and macrophages

**Fig. 1:**
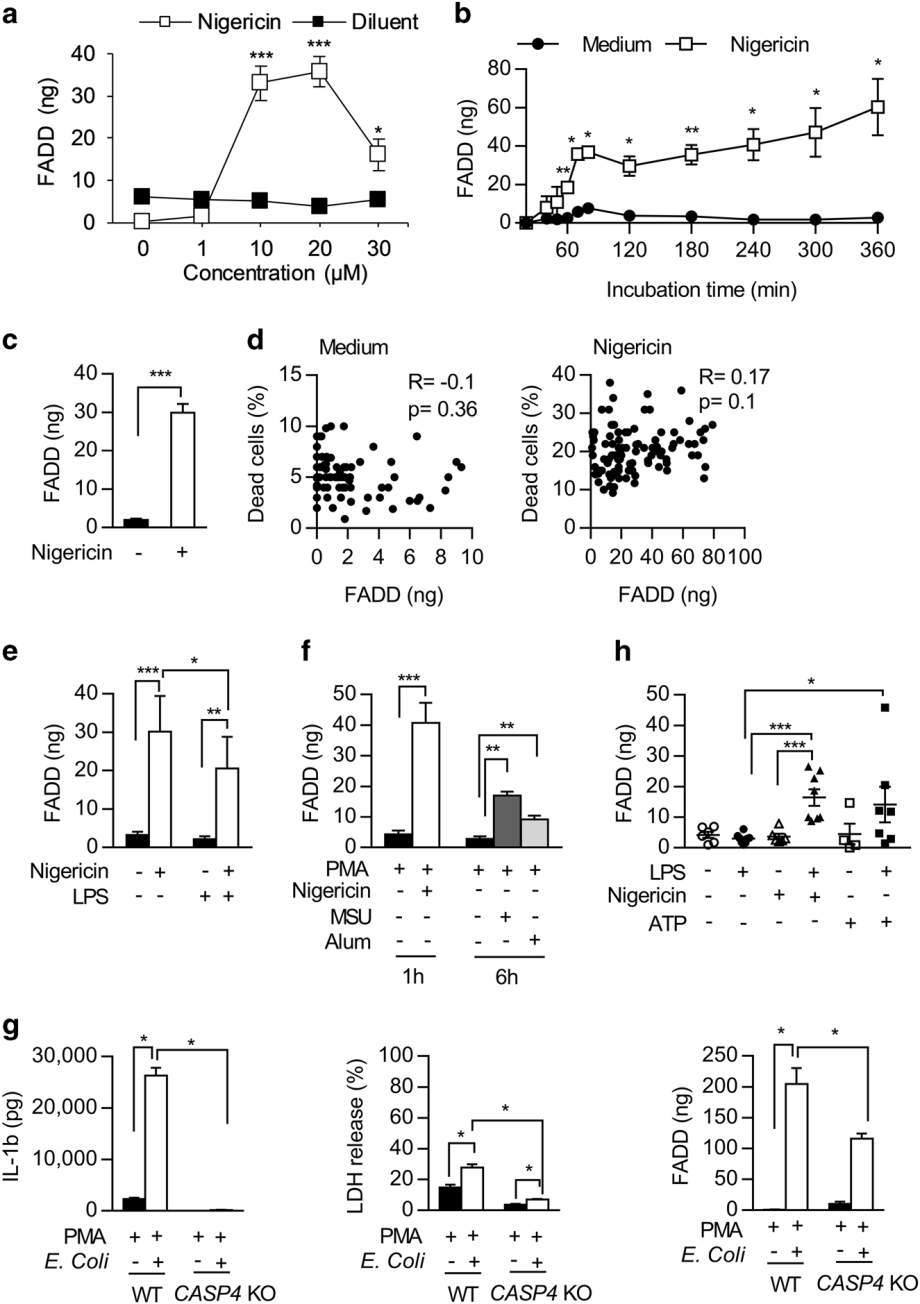
The activation of the NLRP3 inflammasome induces FADD protein secretion from human monocytes. **a–c** ELISA quantification of FADD secreted by (**a**) THP-1 monocytic cell line cultured for 1 h with (open square) or without (closed square) different doses of nigericin (*N* = 6); (**b**) THP-1 cells cultured with (open square) or without (closed circle) 20 µM nigericin during indicated times (*N* = 3–8, depending time point); (**c**) THP-1 cells cultured for 1 h with (+) (*N* = 99) or without (−) (*N* = 93) 20 µM nigericin. **d** Absence of correlation between THP-1 monocyte mortality (evaluated by trypan blue exclusion) and spontaneous (left panel, *N* = 89) and nigericin-induced (right panel, *N* = 95) FADD secretion levels (determined by ELISA). **e**, **f** ELISA quantification of FADD secreted by (**e**) THP-1 cells not primed (−) or primed by 100 ng/ml LPS overnight (+) followed by stimulation 1 h with (+) or without (−) 20 µM nigericin (*N* = 8), and (**f**) PMA-differentiated THP-1 macrophages cultured with (+) or without (−) nigericin (20 µM during 1 h, *N* = 8), MSU (500 µg/ml during 6 h, *N* = 5) or alum (500 µg/ml during 6 h, *N* = 7). **g** ELISA quantification of IL-1β (left) and FADD (right) secreted by PMA-differentiated wild type (WT) or *CASPASE-4* (*CASP4*) KO THP-1 cells cultured with (+) or without (−) *Escherichia coli* (MOI 20 during 2.5 h, *N* = 4), and cell death of these cells (middle) evaluated by LDH assay. **h** ELISA quantification of FADD secreted by PBMCs-derived human primary monocytes from healthy donors not primed (−) or primed by 200 ng/ml LPS overnight (+) followed by stimulation in absence (−) or presence (+) of nigericin (5 µM during 1 h, *N* = 9) or ATP (5 mM during 30 min, *N* = 7); each symbol represents one donor. FADD secretion is depicted as mean ± s.e.m. (**a**–**c**, **e**–**h**). Mann–Whitney test was performed. *P < 0.05, **P < 0.01, ***P < 0.001

We used the THP-1 human monocytic cell line as it is a classical model for inflammasome studies. Nigericin induced dose-dependent and time-dependent secretion of both IL-1β and FADD protein, and cell death, with an optimal effect obtained with 20 µM nigericin (Supplementary Fig. 1a–d and Fig. 1a, b). We observed no significant mortality up to 3 h following nigericin treatment (Supplementary Fig. 1d). After a 6 h-nigericin treatment, protein secretion presumably resulted from cellular content leakage^41^. Accordingly, we chose a one-hour nigericin treatment for further experiments. Although there was a slight correlation between levels of IL-1β secretion and cell death induced by nigericin (Supplementary Fig. 1e-g), no correlation between levels of spontaneous or nigericin-induced FADD secretion and cell death was observed (Fig. 1c, d) arguing against a passive FADD release during cell death. We confirmed that a one-hour nigericin treatment did not induce a significant cell death using propidium iodide staining (Supplementary Fig. 1h). Although LPS-priming of THP-1 cells increased significantly nigericin-induced IL-1β secretion (Supplementary Fig. 1i) it inhibited nigericin-induced FADD secretion (Fig. 1e). Finally, the strongest nigericin-mediated IL-1β (Supplementary Fig. 1j) and FADD (Fig. 1f) secretion were obtained when THP-1 cells were differentiated into macrophages. These results demonstrate that in vitro both monocytes and macrophages release FADD in response to NLRP3 activation by nigericin.

Monosodium urate (MSU) crystals are NLRP3 inducers largely implicated in gout^38^. Since macrophages are more efficient in performing crystal phagocytosis than monocytes, we used PMA-differentiated THP-1 macrophages. MSU crystals induced IL-1β secretion by these cells (Supplementary Fig. 1j). Although less potent than nigericin, MSU significantly induced FADD secretion from THP-1 macrophages (Fig. 1f). Similarly, alum crystals, others NLRP3 inflammasome activators, induced both IL-1β (Supplementary Fig. 1j) and FADD (Fig. 1f) secretion from THP-1 macrophages, without any effect of the diluents used to prepare inflammasome activators (Supplementary Fig. 1k). These results demonstrate that various canonical NLRP3 inflammasome activators increase FADD secretion by THP-1 monocytes/macrophages.

Enteropathogen *Escherichia coli* (EPEC) induced both IL-1β and LDH release from THP-1 macrophages (Fig. 1g). As expected, this release was inhibited in *CASP4*-KO macrophages (Supplementary Fig. 1l and Fig. 1g). Similarly, THP-1 macrophages incubated with EPEC secreted high levels of FADD, and *CASP4*-KO macrophages secreted half less FADD protein than wild type (WT) cells (Fig. 1g). Thus, whereas IL-1β secretion and lytic cell death assayed by LDH release were abrogated in *CASP4*-KO cells, FADD secretion still occurred strongly suggesting that a non-canonical NLRP3 inflammasome inducer activates FADD secretion in human THP-1 macrophages. However, this process only partially requires CASPASE-4.

In primary human peripheral blood mononuclear cells (PBMCs)-derived monocytes from healthy donors (HD), full activation of the canonical NLRP3 inflammasome, consisting in LPS-priming followed by nigericin treatment, induced both IL-1β secretion and cell death (Supplementary Fig. 1m,n). As previously described^13^, LPS alone activated the alternative NLRP3 inflammasome, leading to a lower but substantial IL-1β secretion (Supplementary Fig. 1m). Human primary monocytes spontaneously secreted low levels of FADD, without any increase upon LPS or nigericin treatment (Fig. 1h) suggesting that the alternative NLRP3 inflammasome was not engaged in FADD secretion. However, priming of primary monocytes with LPS followed by nigericin treatment increased FADD secretion at a level close to that observed with THP-1 cells (Fig. 1h). Primary monocytes also secreted FADD when ATP, a classical damaged-associated molecule activating NLRP3 inflammasome, replaced nigericin (Fig. 1h).

### FADD secretion is under the control of the NLRP3 inflammasome

**Fig. 2:**
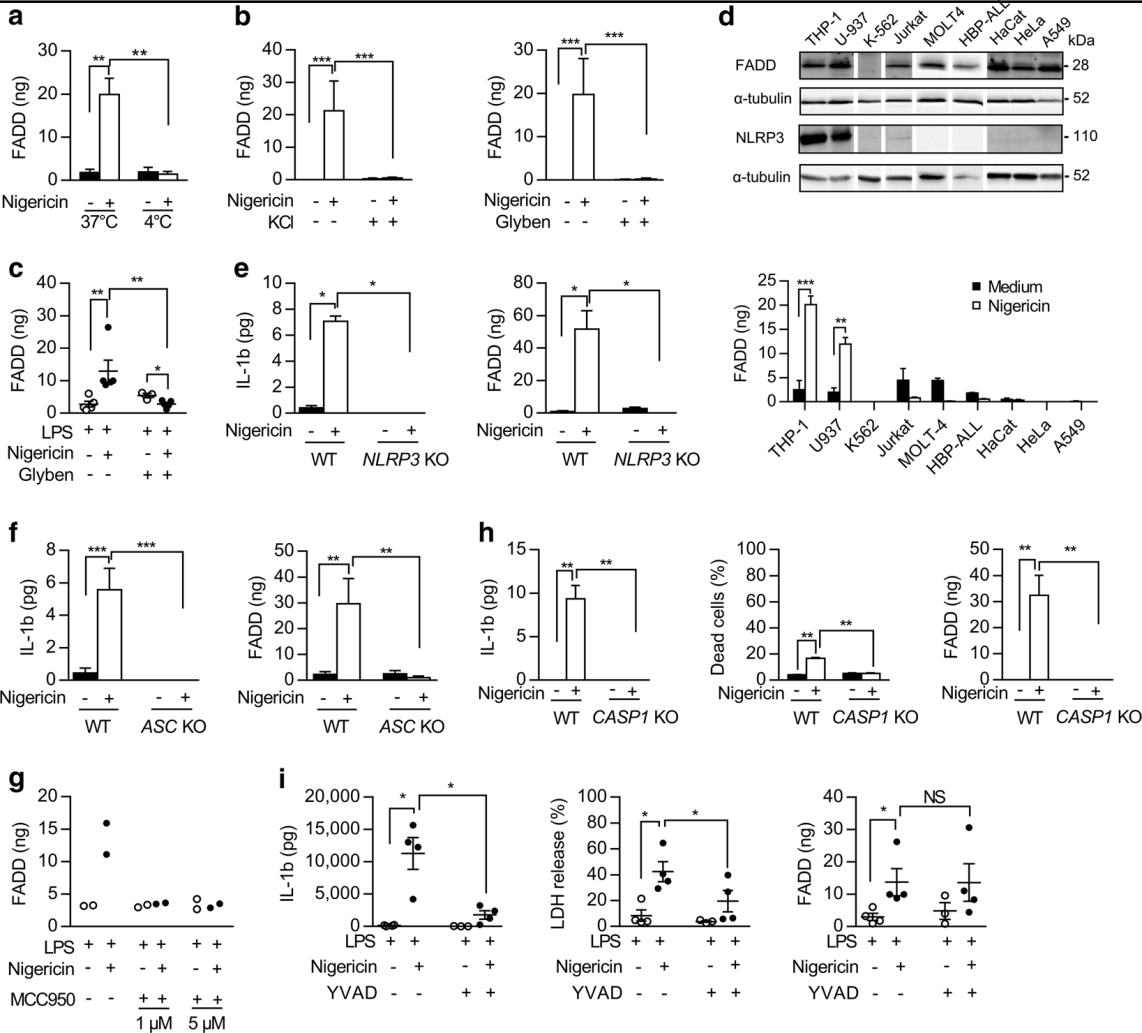
FADD secretion requires potassium efflux, NLRP3 sensor, ASC adaptor, and CASPASE-1 molecule. **a–c** ELISA quantification of FADD secreted by (**a**) THP-1 cells cultured for 1 h with (+) or without (−) 20 µM nigericin at 37 °C or 4 °C (*N* = 5); (**b**) THP-1 cells cultured for 1 h with (+) or without (−) 20 µM nigericin in absence (−) or presence (+) of potassium efflux inhibitors (130 mM KCl, *N* = 6–10; 50 µM glybenclamide, *N* = 6–12); (**c**) PBMCs-derived monocytes from healthy donors primed by LPS (200 ng/ml overnight) followed by 1 h stimulation with (+) or without (−) 5 µM nigericin in absence (−) or presence (+) of 50 µM potassium efflux inhibitor (*N* = 3–5); each symbol represents one donor. **d** Upper panel: western blot analysis of FADD and NLRP3 proteins in myeloid (THP-1, U937, K562), lymphoid (Jurkat, MOLT-4, DND-41, HBP-ALL) and non-hematopoietic (HeLa, HaCat, A549) cell lines; α-tubulin serves as a loading control. Lower panel: ELISA quantification of FADD secreted by these cell lines cultured for 1 h with (open bars) or without (closed bars) 20 µM nigericin (*N* = 4). **e–i** ELISA quantification of IL-1β and FADD secreted by (**e**) wild type (WT) or *NLRP3* sensor knockout (*NLRP3* KO) THP-1 cells cultured for 1 h with (+) or without (−) 20 µM nigericin (*N* = 5); (**f**) WT or *ASC* adaptor knockout (*ASC* KO) THP-1 cells cultured for 1 h with (+) or without (−) 20 µM nigericin (*N* = 5); (**g**) PBMCs-derived monocytes from healthy donors primed by LPS (200 ng/ml overnight) were pretreated for 1 h in absence (−) or in presence (+) of 1 or 5 µM of the NLRP3 inflammasome inhibitor MCC950, followed by 1 h stimulation in absence (−) or presence (+) of 5 µM nigericin (each symbol represents one donor); (**h**) WT or *CASPASE-1* knockout (*CASP1* KO) THP-1 cells cultured for 1 h with (+) or without (−) 20 µM nigericin (*N* = 6); cell death of these cells was evaluated by trypan blue exclusion; (**i**) PBMCs-derived monocytes from healthy donors primed by LPS (200 ng/ml overnight) followed by 1 h stimulation in absence (−) or presence (+) of 5 µM nigericin in absence (−) or presence (+) of 10 µM of the CASPASE-1 chemical inhibitor Ac-YVAD-cmk (*N* = 4), and cell death evaluated by LDH release from the same PBMCs-derived monocytes; each symbol represents one donor. In all panels, results are expressed as mean ± s.e.m. Mann–Whitney test was performed, *P < 0.05, **P < 0.01, ***P < 0.001. NS, not significant

Nigericin-induced FADD secretion was completely inhibited at 4 °C (Fig. 2a) demonstrating that FADD secretion was an active process. Blocking K^+^ efflux by incubating THP-1 monocytes with either high K^+^ concentration or glybenclamide^42^ completely blocked nigericin-induced IL-1β (Supplementary Fig. 2a) and FADD secretion (Fig. 2b). In human primary monocytes, glybenclamide inhibited both IL-1β and FADD secretion and decreased LDH release induced by LPS-priming and nigericin treatment (Fig. 2c and Supplementary Fig. 2b,c). Thus, nigericin-induced K^+^ efflux is essential for FADD secretion both in vitro and ex vivo.

To test whether FADD secretion depends upon NLRP3 inflammasome, we examined FADD secretion in several cell lines expressing different levels of the NLRP3 protein (Fig. 2d). All these cells expressed FADD except the K-562 cell line, used as a negative control. Only THP-1 and U-937 myeloid cells expressed strong levels of NLRP3. Although most cell lines secreted FADD spontaneously, only THP-1 and U-937 cells secreted significant levels of FADD in response to nigericin (Fig. 2d), demonstrating that nigericin-induced FADD secretion was correlated with NLRP3 protein expression.

To confirm this result, we generated THP-1 cells invalidated for NLRP3 or ASC. *ASC*-KO THP-1 cells still expressed the NLRP3 inflammasome inhibitory c isoform of ASC, but did not express the pro-inflammatory a and b isoforms^43^ (Supplementary Fig. 2d). In contrast to WT cells, neither *NLRP3*-KO nor *ASC*-KO THP-1 cells could secrete IL-1β or FADD in response to nigericin (Fig. 2e, f) demonstrating that both NLRP3 and ASC were essential for FADD secretion. In primary monocytes from HD, the NLRP3 inflammasome-specific inhibitor MCC950 completely inhibited LPS/nigericin-induced IL-1β secretion and LDH release (Supplementary Fig. 2e). Moreover, MCC950 abrogated FADD secretion following LPS/nigericin treatment (Fig. 2g) confirming that the NLRP3 inflammasome triggered FADD secretion in human primary monocytes.

Both IL-1β processing and secretion and pyroptosis are downstream consequences of CASPASE-1 activation^18^. In contrast with WT cells, *CASP1*-KO THP-1 cells (Supplementary Fig. 2d) did not secrete IL-1β nor died in response to nigericin (Fig. 2h). Moreover, the knock down of *CASP1* completely inhibited nigericin-induced FADD secretion (Fig. 2h). In human primary monocytes, CASPASE-1 blockade using Ac-YVAD-cmk inhibited IL-1β secretion and LDH release following LPS/nigericin treatment (Fig. 2i). In contrast, Ac-YVAD-cmk did not affect nigericin-induced FADD secretion (Fig. 2i). These results are in accordance with an enzymatic activity-independent role of CASPASE-1 in FADD secretion.

### FADD secretion is an active process requiring extracellular glucose

**Fig. 3:**
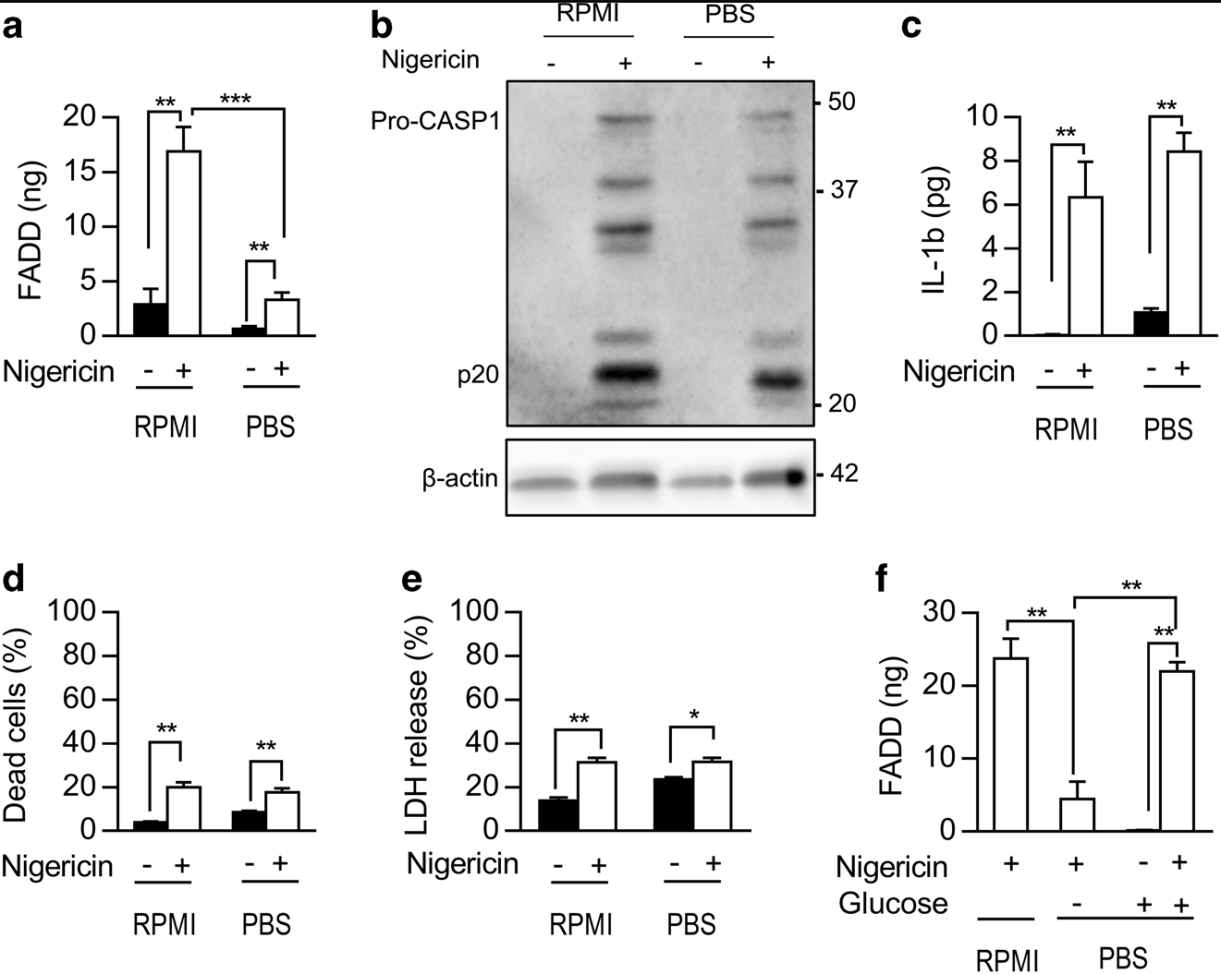
FADD secretion is an active process requiring extracellular glucose. **a** ELISA quantification of FADD secreted by THP-1 cells cultured for 1 h with (+) or without (−) 20 µM nigericin diluted in RPMI 1640 medium or in PBS buffer (*N* = 6). **b** Western blot analysis of CASPASE-1 protein in the culture supernatant from THP-1 cells cultured for 1 h with (+) or without (−) 20 µM nigericin in RPMI 1640 medium or PBS buffer. β-actin serves as a loading control. Pro-CASP1, pro-CASPASE-1; p20, cleaved form of CASPASE-1. Results are representative of three independent experiments. **c** ELISA quantification of IL-1β secreted by THP-1 cells cultured for 1 h with (+) or without (−) 20 µM nigericin diluted in RPMI 1640 medium or in PBS buffer (*N* = 6). **d**, **e** Cell death evaluated by trypan blue exclusion (**d**) and LDH release assay (**e**), from THP-1 monocytes cultured for 1 h with (+) or without (−) 20 µM nigericin diluted in RPMI 1640 medium or in PBS buffer (*N* = 6). **f** ELISA quantification of FADD secreted by THP-1 cells cultured for 1 h with (+) or without (−) 20 µM nigericin diluted in RPMI 1640 medium or in PBS buffer in absence (−) or presence (+) of 2 mg/ml of glucose, which is equivalent to the glucose concentration in RPMI 1640 medium (*N* = 6). Results are expressed as mean ± s.e.m. (**a**, **c**–**f**). Mann–Whitney test was performed, *P < 0.05, **P < 0.01, ***P < 0.001. RPMI, RPMI 1640 medium

In contrast to what we observed in RPMI 1640 medium, FADD secretion was strongly inhibited in PBS buffer (Fig. 3a). However, nigericin activated the NLRP3 inflammasome in THP-1 monocytes in both RPMI 1640 medium and PBS buffer, as attested by the presence of cleaved CASPASE-1 in the culture supernatants from these cells (Fig. 3b). Confirming these data, nigericin induced both IL-1β secretion and cell death independently of the culture medium used (Fig. 3c–e and Supplementary Fig. 2f). Whereas addition of calcium and magnesium did not restore FADD secretion (Supplementary Fig. 2f), glucose addition to the PBS buffer restored nigericin-induced FADD secretion (Fig. 3f and Supplementary Fig. 2g). Although inflammasome activation ultimately leads to pyroptosis, resulting to unspecific release of most cellular proteins^41^, our results demonstrate that FADD secretion is an active and regulated process requiring extracellular glucose.

### FADD protein unconventional secretion occurs upon microvesicle shedding

**Fig. 4:**
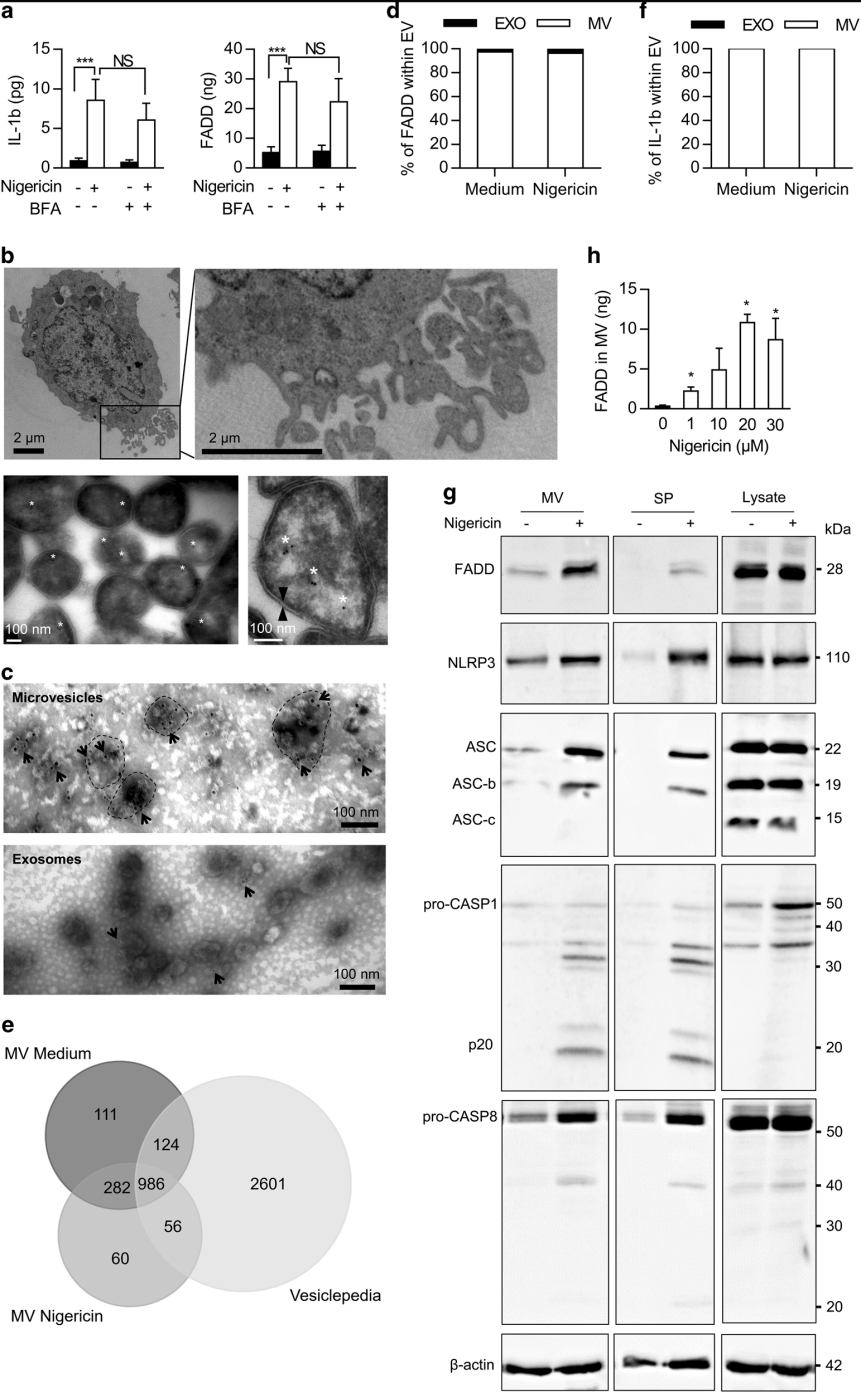
FADD unconventional secretion occurs through microvesicles shedding. **a** ELISA quantification of IL-1β and FADD secreted by THP-1 monocytes cultured for 1 h with (+) or without (−) 20 µM nigericin in absence (−) or presence (+) of 5 µg/ml of the ER–Golgi transport inhibitor brefeldin A (*N* = 3). **b** Upper panels: left, electron microscopy analysis of one THP-1 cell spontaneously shedding microvesicles; right, ×4 magnification of area outlined at left. Lower panel: FADD-specific immunogold electron microscopy of extracellular vesicles spontaneously released by THP-1 monocytes. Asterixs indicate gold particles (FADD-specific labeling); closed triangles indicate plasma membrane. **c** FADD-specific immunogold electron microscopy of microvesicles (isolated by 10,000×*g* centrifugation) and exosomes (isolated by 100,000×*g* centrifugation) spontaneously released by THP-1 monocytes. Microvesicles are surrounded by dotted lines. Arrows indicate gold particles (FADD-specific labeling). **d** ELISA quantification of FADD contained within extracellular vesicles (EV) including microvesicles (MV) and exosomes (EXO) secreted by THP-1 monocytes following 1 h incubation with (Nigericin) or without (Medium) 20 µM nigericin (*N* = 7). **e** Venn diagram of proteins identified by LC–MS/MS analysis in microvesicles derived from untreated (MV Medium) or 1 h nigericin-treated (MV Nigericin) THP-1 cells compared to the Vesiclepedia database of MV proteins. **f** ELISA quantification of IL-1β contained within extracellular vesicles (EV) including microvesicles (MV) and exosomes (EXO) secreted by THP-1 monocytes following 1 h incubation with (Nigericin) or without (Medium) 20 µM nigericin (*N* = 7). **g** Western blot analysis of FADD protein and inflammasome components contained within microvesicles (MV, isolated from supernatant from 15 × 10^6^ cells by 10,000×*g* centrifugation), soluble proteins (SP, obtained from supernatant from 15 × 10^6^ cells by 100,000×*g* centrifugation), and within THP-1 cells (Lysate) following 1 h culture with (+) or without (−) 20 µM nigericin. β-actin serves as a loading control. Pro-CASP1, pro-CASPASE-1; p20, cleaved form of CASPASE-1; pro-CASP8, pro-CASPASE-8. Results are representative of three independent experiments. **h** Culture supernatants from THP-1 monocytic cell line cultured for 1 h with different doses of nigericin, as indicated, were treated with or without Tween 20 to induce MV burst. Then ELISA quantification of FADD was performed. Histograms represent the difference between FADD quantities detected with and without Tween 20, which indicates the quantity of FADD enclosed within MV (*N* = 4). Results are expressed as mean ± s.e.m. (**a**, **d**, **f**, **h**). Mann–Whitney test was performed, *P < 0.05, **P < 0.01, ***P < 0.001. NS, not significant

Most of the signal peptide prediction software identified FADD as leaderless protein, and some of them predicted FADD unconventional secretion (Table 1 and Supplementary Table 1). Brefeldin A (BFA), an ER-Golgi transport inhibitor, slightly decreased nigericin-induced IL-1β secretion from THP-1 monocytes (Fig. 4a and Supplementary Fig. 3a). Accordingly, nigericin-induced FADD secretion was resistant to BFA (Fig. 4a and Supplementary Fig. 3a) suggesting that FADD was secreted by unconventional secretion pathway.

**Table 1:** Lack of peptide signal in the human FADD protein sequence

Software	Signal peptide prediction	Protein secretion prediction	Reference
Phobius	Yes	–	^66^
Signal-3L	No	–	^67^
Signal-BLAST	No	–	^68^
SignalP	No	–	^69^
Predotar	No	–	^70^
Spoctopus	No	–	^71^
PrediSi	Yes^a^	Yes	^72^
Secretome P	No	Yes/UPS	^73^

– Not determined

^a^Score > 0.50: sequence very likely contains a signal peptide; human FADD score: 0.52

THP-1 cells spontaneously shed plasma membrane-derived microvesicles ranging ~100–1000 nm (Fig. 4b upper panel). Using FADD-specific immunogold electron microscopy, we showed that these vesicles enclosed the FADD protein (Fig. 4b lower panel). We next separated extracellular vesicles (EVs) into the microvesicles derived from the plasma membrane (Supplementary Fig. 3b) and the exosomes (50–100 nm) (Supplementary Fig. 3c). Nigericin treatment increased the release of both types of vesicles (Supplementary Fig. 3b, c). We detected both FADD-containing vesicles and soluble FADD proteins in the microvesicle-enriched fraction (Fig. 4c upper panel). In contrast, exosomes did not contain FADD (Fig. 4c lower panel) as confirmed by ELISA quantification (Fig. 4d). Moreover, proteomics analysis showed that 75% of proteins identified in microvesicles derived from both untreated (1110/1503) and nigericin-treated (1042/1384) THP-1 cells overlapped with the Vesiclepedia database of microvesicle proteins (http://microvesicles.org/)^44^ (Fig. 4e). Among these proteins, we detected most of the typical markers of microvesicles^32^, including CD63 tetraspanin, ALIX, and TSG101 ESCRT components, and ARF6 membrane transport molecule (Table 2). In addition, microvesicles contained IL-1β (Fig. 4f), native and processed CASPASE-1, ASC, and NLRP3 proteins (Fig. 4g). CASPASE-8 is a main partner of FADD in most signaling pathways and participates along with FADD to the canonical, non-canonical, and alternative NLRP3 inflammasome^12,13^. As expected, microvesicles also contained CASPASE-8 (Fig. 4g). Moreover, activation of the NLRP3 inflammasome by nigericin increased levels of FADD, CASPASE-1, ASC, NLRP3, and CASPASE-8 in both microvesicles and soluble fractions, whereas intracellular FADD levels were not affected (Fig. 4g and Supplementary Fig. 3d). Secreted FADD was detected as a full-length protein following nigericin treatment (Fig. 4g). To evaluate the proportion of FADD within microvesicles, we treated the culture supernatant from nigericin-stimulated THP-1 monocytes with tween 20 to break microvesicles, or we left the culture medium untreated to preserve microvesicles integrity. After 1 h nigericin treatment with 20 µM, up to 10 ng of FADD was contained within microvesicles (Fig. 4h), preventing its detection by ELISA (Supplementary Fig. 3e). In contrast, only very little FADD was detected in the microvesicle fraction (around 0.5 ng, Supplementary Fig. 3f) when the culture supernatant was isolated by centrifugation instead of using the trans-well system. In this case, we detected most of the FADD proteins in the soluble proteins fraction (Supplementary Fig. 3f), suggesting that centrifugation induced most microvesicle lysis. We obtained similar results looking at IL-1β (Supplementary Fig. 3f). All these results suggest that monocytes might secrete an important part of the FADD, IL-1β, and NLRP3 inflammasome components through microvesicle shedding.

**Table 2:** Insight of proteins detected in FADD-containing microvesicles released by THP-1 monocytes

Family of proteins		Protein ID	Protein name	Presence in MV^a^
Monocytes markers		P08571	CD14	+
		P20702	CD11c	+
Adhesion molecules	Tetraspanins	P08962	CD63	+
		P60033	CD81	+
		P21926	CD9	−
	Integrins	P13612	α4integrin	+
		P20701	LFA-1	+
		Q08431	MFGE8/ Lactadherin	−
Membrane transport/fusion (lipid bound)		P07355	Annexin A2	+
		P08758	Annexin A5	+
		O75955	Flotillin-1	+
		Q14254	Flotillin-2	+
		P20339	RAB5A	+
		P51148	RAB5C	+
		P51149	RAB7A	+
		Q15907	RAB11B	+
		Q15286	RAB35	+
		P62330	ARF6 (ADP-ribosylation factor)	+
Other transmembrane proteins		P11279	LAMP-1	+
		P02786	Transferrin receptor protein 1	+
Antigen presentation		P01892,P30464,P04222	HLA class I antigens	+
		P04229,Q30154,P01920	HLA class II antigens	−
Endosomal sorting complex required for transport (ESCRT) components		Q8WUM4	ALIX	+
		Q99816	TSG101	+
		O75351	VPS4B	+
Signal transduction		P62258	14–3–3 protein epsilon	+
		O00560	Syntenin-1	+
Enzymes		P68104	Elongation factor 1-alpha	+
		P13639	Elongation factor 2	+
		P04406	GAPDH	+
Cytoskeletal proteins		P60709	Actin	+
		P23528	Cofilin	+
		P26038	Moesin	+
		P07437	Tubulin	+
Other cytosolic proteins		P62805	Histones H4	+
		P62249	40S ribosomal protein S16	+
		P49720	Proteasome subunit beta type-3	+

^a^Microvesicles (MV) were isolated by 10,000×*g* centrifugation. Results are representatives of microvesicles from both untreated and nigericin-treated THP-1 cells

*ALIX* ALG-2-interacting protein X, *GAPDH* glyceraldehyde-3-phosphate dehydrogenase, *HLA* human leukocyte antigen, *LAMP* lysosome-associated membrane glycoprotein, *LFA* leukocyte function-associated, *MFGE8* milk fat globule-epidermal growth factor-factor 8, *RAB* Ras-related protein, *TSG101* tumor susceptibility gene 101 protein, *VPS* vacuolar protein sorting-associated protein

### FADD protein secretion occurs during inflammatory diseases

**Fig. 5:**
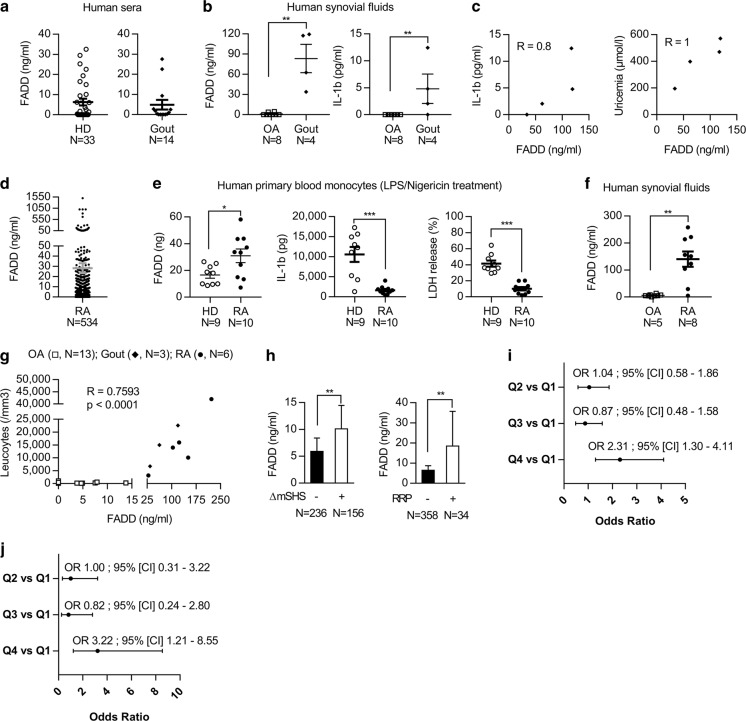
FADD secretion occurs in vivo and is a local marker of inflammation. **a** ELISA quantification of FADD in the sera from healthy donors (HD) provided by the Etablissement Français du Sang and in the sera from gout attack-suffering patients (Gout). **b** ELISA quantification of FADD and IL-1β in the synovial fluids from osteoarthritis (OA) and gout attack-suffering patients (Gout). **c** Spearman correlation between FADD and IL-1β concentrations in the synovial fluids from gout attack-suffering patients (left), and spearman correlation between synovial FADD levels and uricaemia of gout attack-suffering patients (right). **d** ELISA quantification of FADD in the sera from rheumatoid arthritis (RA) suffering patients of the ESPOIR cohort at inclusion. ESPOIR consortium cohort: untreated recently diagnosed arthritis suffering patients. 2010 ACR/EULAR classification criteria for RA was applied. **e** ELISA quantification of FADD and IL-1β secreted by PBMCs-derived monocytes from healthy donors (HD) and rheumatoid arthritis suffering patients (RA) primed by LPS (200 ng/ml overnight) followed by 1 h stimulation in the presence of 5 µM nigericin; and cell death evaluated by LDH assay from the same cells. **f** ELISA quantification of FADD in the synovial fluids from osteoarthritis (OA; *N* = 8 samples corresponding to five different patients) and rheumatoid arthritis (RA) suffering patients. Synovial fluids from two OA patients were aspirated at two and three different times, respectively. **g** Spearman correlation between FADD concentration (determined in (**b**) and (**f**)) and leukocytes number in the synovial fluids from OA, gout, and RA patients. **h** FADD serum levels of patients from the ESPOIR cohort at inclusion were associated with structural progression of early RA defined by an increase of van der Heijde-modified Sharp score (ΔmSHS) (*N* = 392). Radiographic progression was defined as an increase in mSHS > 1 (+), assessed between baseline and the end of years 1 and 2 of follow-up. Rapid radiographic progression (RRP) was defined as an increase in mSHS > 10 (+) within the first 2 years of follow-up (i.e., 5 points per year). Results are expressed as median ± 95% CI. **i** Risk of structural progression and **j** Risk of rapid radiographic progression, in early RA patients from the ESPOIR cohort within the first 2 years of follow-up by quartiles (Q) of serum FADD level at inclusion. Q1, <0.6; Q2, 0.6–7.2; Q3, 7.2–19.1, and Q4, >19.1 ng/ml. Results are expressed as odds ratio (OR) ± 95% confidence intervals (CI). Each symbol represents one donor/patient (**a–g**). Bar represents mean ± sem (**a**, **b**, **d–f**). Mann–Whitney test was performed, *P < 0.05, **P < 0.01, ***P < 0.001

Gout attack is the best-characterized NLRP3-dependent autoinflammatory disease^38,39^. FADD concentration in the sera from HD was low, and we measured similar levels of FADD in the sera from patients having gout attack (Fig. 5a). However, we detected high concentrations of both FADD and IL-1β in the knee synovial fluid from patients with gout attack, as compared to patients with osteoarthritis (OA), considered as a non-inflammatory rheumatic disease (Fig. 5b). Gout attack occurs upon deposition of MSU crystals in the tissue that induce IL-1β release. Pertinently, synovial fluid levels of FADD and IL-1β were correlated (Fig. 5c). MSU crystals are NLRP3 inducers formed when concentrations of uric acid increase in the body. We found that FADD concentrations in the joint correlated with uricaemia (Fig. 5c). Altogether, these data suggest that FADD secretion in the synovial fluid from gout patients might result from NLRP3 inflammasome activation by MSU crystals.

As NLRP3 inflammasome activation is implicated in RA pathophysiology^40,45^, we measured FADD in the sera from RA-suffering patients of the ESPOIR cohort (Etude et Suivi des Polyarthrites Indifférenciées Récentes)^46^, a French national multi-centric cohort of patients having early arthritis that started <6 months previously. Elevated FADD concentration in the sera from RA patients, reaching 1503 ng/ml, was detected (Fig. 5d). Consequently, we examined FADD secretion by PBMCs-derived monocytes from RA patients. LPS-primed and nigericin-treated primary monocytes from RA patients secreted elevated levels of FADD compared to HD monocytes (Fig. 5e). Surprisingly, IL-1β secretion was strongly inhibited as compared to HD monocytes (Fig. 5e). Accordingly, IL-1β levels in the sera from RA patients of the ESPOIR cohort were almost zero (mean IL-1β = 0.22 pg/ml, *N* = 398). Concomitantly, lytic cell death of RA monocytes was strongly inhibited as compared to HD monocytes (Fig. 5e). Thus, in RA monocytes, IL-1β secretion and LDH release were inhibited, whereas FADD secretion was exacerbated in response to NLRP3 inflammasome activation. Moreover, we measured high concentrations of FADD in the knee synovial fluid from RA-suffering, but not from OA-suffering patients (Fig. 5f). Thus, FADD secretion occurred both systemically in the sera and locally in the joint from RA patients.

FADD levels in synovial fluids from OA-suffering, RA-suffering, and gout-suffering patients positively correlated with the inflammatory status of the joint evaluated by the number of leukocytes (Fig. 5g), strongly suggesting that soluble FADD could constitute a new biomarker of inflammation in rheumatic diseases. Moreover, soluble FADD appeared as a biomarker of structural severity in early RA-suffering patients from the ESPOIR cohort, since baseline serum FADD levels were associated with structural progression (characterized by joint erosion and joint space narrowing) within the first 2 years of follow-up. Indeed, baseline serum FADD levels were higher among RA patients with than without radiological progression (Fig. 5h, left panel). Furthermore, levels of FADD in the sera from RA patients from the ESPOIR cohort correlated with a rapid radiographic progression (RRP) (Fig. 5h, right panel), indicating a more severe disease. When considering FADD distribution by quartiles, FADD level in the upper quartile (>19.1 ng/ml) was associated with risk of structural progression (Fig. 5i) and rapid structural progression (Fig. 5j) as compared with the lowest quartile (<0.6 ng/ml). These results suggest that baseline serum FADD levels may be a strong determinant of structural damage among patients with early RA.

## Discussion

FADD is a pleiotropic molecule playing key role in numerous cellular processes including several forms of cell death, proliferation, innate immunity, and inflammation. Patients with a homozygous missense mutation in *FADD* die early in childhood from infectious syndrome^7^, and lack of FADD leads to acute sensitivity to several viruses in mice. The FADD antiviral activity involves a FADD–RIPK1–Tank-binding kinase 1 (TBK1) complex named the innateosome, which leads to interferon production to mediate antiviral responses^3^. A similar FADD-dependent mechanism is implicated in defense against bacteria in Drosophila, suggesting that FADD requirement in innate immunity is evolutionarily conserved^47^. In this line of thinking, it was shown that FADD contributes to the canonical, non-canonical, and alternative NLRP3 inflammasome signaling pathways leading to IL-1β secretion, an inflammatory cytokine playing a key role in innate immunity^12,13,25,48^.

**Fig. 6:**
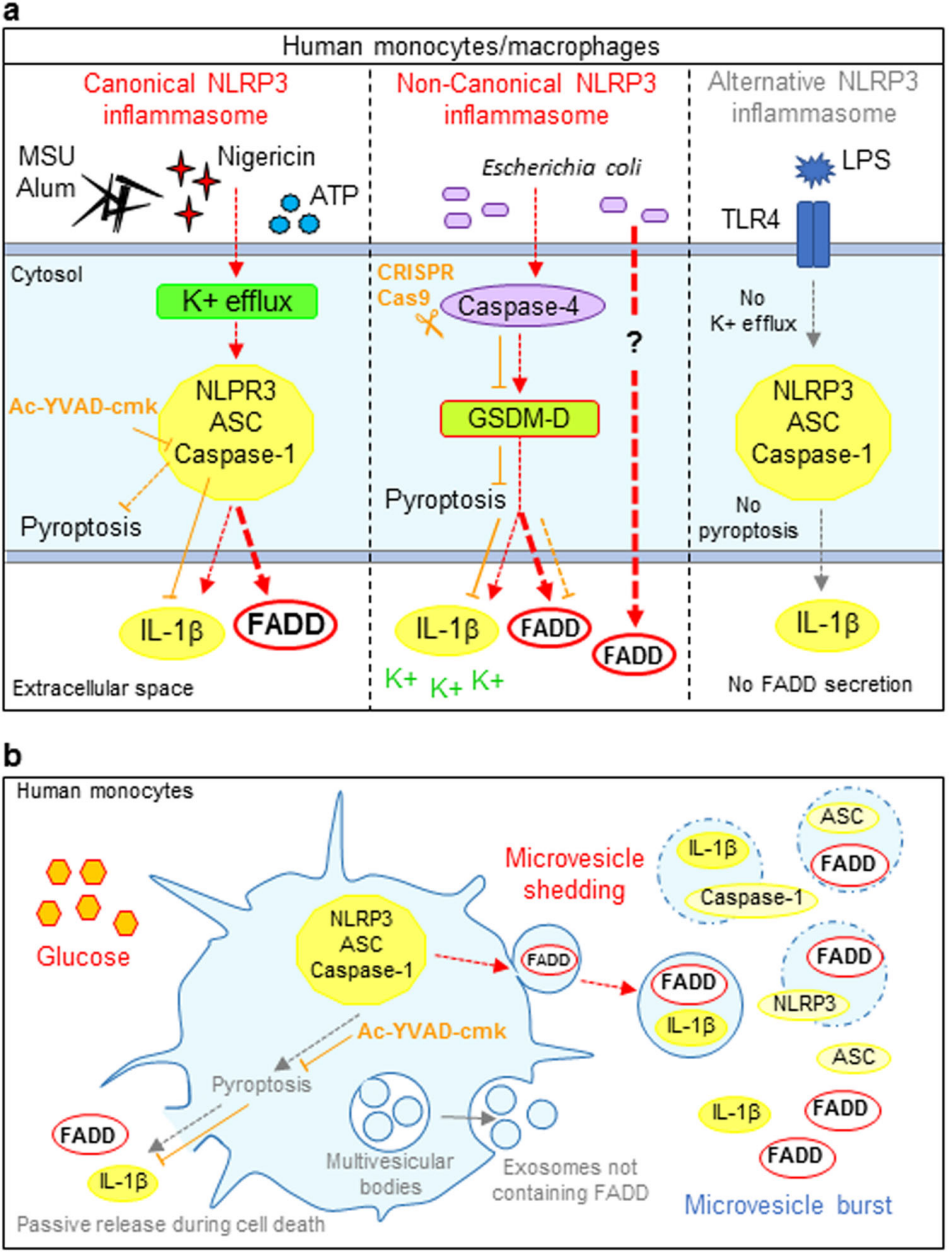
FADD protein unconventional secretion is under the control of the NLRP3 inflammasome and occurs upon several pathways in human monocytes. **a** Canonical (nigericin, ATP, MSU, and alum crystals) and non-canonical (enteropathogen *Escherichia coli*), but not alternative (LPS), NLRP3 inflammasome inducers activate FADD secretion in human monocytes/macrophages. *Left*: FADD secretion through canonical NLRP3 inflammasome requires K^+^ efflux, NLRP3 sensor, ASC adaptor, and CASPASE-1 molecule. Cell death, which is partially inhibited by CASPASE-1 inhibitor (Ac-YVAD-cmk), accompanies the secretion process. Ac-YVAD-cmk totally inhibited IL-1β but not FADD release, suggesting that FADD secretion might require no or little CASPASE-1 enzymatic activity. *Middle*: Non-canonical NLRP3 inflammasome requires CASPASE-4 to cleave GSDMD. Activated GSDMD forms pores in the membrane leading to cellular contents release—creating a K^+^ efflux that in turn activates the NLRP3 inflammasome—and cell death. Invalidating *CASPASE-4* decreased by half FADD release, suggesting that FADD secretion might occur through two distinct pathways. *Right*: Alternative NLRP3 inflammasome induces IL-1β but not FADD secretion. **b** FADD, like IL-1β, is a leaderless protein unconventionally secreted by human monocytes through several pathways following canonical NLRP3 inflammasome activation. IL-1β can be secreted by passive release during cell death (pyroptosis). This pathway accounts for FADD secretion, particularly in the late stage of NLRP3 inflammasome activation. Nevertheless, FADD can also be secreted through plasma membrane-derived microvesicle shedding. Microvesicles also contain IL-1β, native and processed CASPASE-1, ASC and NLRP3 proteins. All these proteins could be delivered to the extracellular space upon microvesicle burst. In human THP-1 monocytes, FADD and IL-1β unconventional secretion does not occur through exosome release. Moreover, FADD (but not IL-1β) secretion process requires extracellular glucose

In the current study, we reported that NLRP3 inflammasome activation by several stimuli, including the bacterial toxin nigericin, MSU and alum crystals, extracellular ATP, and live pathogen bacteria-induced FADD secretion from human monocytes and macrophages (Fig. 6a). The clinical significance of our findings were illustrated by our observation that high levels of FADD in the synovial fluids from gout patients were correlated with levels of NLRP3 inflammasome inducer (MSU derived from high uricaemia) and NLRP3 inflammasome activity (IL-1β concentrations), confirming that NLRP3 inflammasome activation might lead to FADD secretion in vivo. However, mechanisms underlying IL-1β and FADD secretion processes partially differed. FADD secretion by THP-1 monocytes required glucose in the culture medium whereas IL-1β secretion occurred in the absence of extracellular glucose. Moreover, whereas knockout of *CASP4* abrogated IL-1β secretion, it only inhibited by half the secretion of FADD following activation of the non-canonical NLRP3 inflammasome. A similar mechanism was described for IL-18. While dendritic cells from *Caspase-11*-deficient mice do not secrete IL-1β in response to the activation of the non-canonical NLRP3 inflammasome, they still secrete IL-18^49^. However, the mechanism accounting for this IL-18 secretion is unknown. Furthermore, although LPS alone (alternative pathway) induced IL-1β secretion from primary human monocytes, it did not induce FADD secretion (Fig. 6a).

NLRP3 inflammasome activation results in GSDMD-induced pore formation^29,50,51^ and consequent cell lysis, leading to protein leakage in the extracellular space. FADD is presumably released by this way when NLRP3 inflammasome activation lasts for several hours. However, no correlation between secreted FADD and cell death levels was found. Moreover, we found that FADD secretion occurs in the absence of IL-1β secretion and LDH release (as observed for *CASP4*-KO THP-1 macrophages and primary RA monocytes) and, reciprocally, IL-1β secretion and LDH release occurs in the absence of FADD secretion (as observed for THP-1 monocytes in PBS buffer). Thus, FADD secretion in response to NLRP3 inflammasome activation is an active and regulated process. Indeed, FADD, but not IL-1β, required glucose for its secretion (Fig. 6b). One hypothesis is that glucose may be necessary for the sorting of FADD into vesicles.

UPS means that proteins bypass the classical ER–Golgi trafficking to be secreted^27,52^. We described FADD as a new member of the family of unconventionally secreted proteins. Indeed, we identified FADD as a leaderless protein and microvesicle shedding, but not exosome release, as a potential mechanism accounting for FADD UPS (Fig. 6b). Cells dying by pyroptosis undergo membrane blebbing before rupture of the plasma membrane. They produce bubble-like protrusions called pyroptotic bodies, ranging from 1 to 5 µm^30^. However, FADD-containing microvesicles sized predominantly between 200 and 400 nm, and expressed most of the typical markers of microvesicles. This excludes the possibility that FADD-containing microvesicles were pyroptotic bodies. All our results suggest that at early time following inflammasome activation, most of the FADD secretion occurs through microvesicle shedding. Thereafter, microvesicle burst, but not cell lysis, may lead to high levels of extracellular soluble FADD (Fig. 6b) as previously described in mouse thyroid cells^35^. Nonetheless, the hypothesis that FADD secretion could occur through another UPS pathway leading to direct proteins release could not be ruled out^28^.

We showed that FADD secretion occured during inflammatory diseases. NLRP3 inflammasome is implicated in the production of IL-1β, an important mediator of cartilage destruction^53^. Nevertheless, IL-1β levels in the sera from RA-patients were almost zero and did not correlate with bone erosion. By contrast, FADD levels in the sera from RA-suffering patients of the ESPOIR cohort correlated with disease severity defined by structural damage. Moreover, FADD levels in synovial fluids correlated with the inflammatory status of the joint. An intriguing question is whether secreted FADD is just a biomarker of inflammation or whether it might play a role in the inflammatory process. Notably, CASPASE-1, ASC, and NLRP3 were secreted concomitantly to the FADD protein. Secreted components of the inflammasome can form an extracellular oligomeric complex whose role is to amplify the inflammatory response^36,37^. FADD is a component of the NLRP3 inflammasome. FADD, like ASC^54^, can oligomerize into filaments^55^. Thus, we can hypothesize that FADD, like NLRP3 and ASC, might be secreted as a particulate danger signal participating to the extracellular inflammasome complex. In this case, increase of extracellular FADD might contribute to RA pathogenesis, by exacerbating and propagating the inflammatory response.

In conclusion, our results advance our understanding of the mechanisms contributing to FADD protein regulation in human monocytes/macrophages and provide some important clues on its potential clinical relevance in chronic rheumatic diseases such as gout or RA.

## Materials and methods

### Cell culture

The human myeloid THP-1, U-937, and K-562, and lymphoid Jurkat, MOLT-4, and HBP-ALL cell lines were cultured in RPMI 1640 with GlutaMAX (Gibco); the human epithelial HeLa and keratinocyte HaCat cell lines were cultured in DMEM with GlutaMAX (Gibco); the human epithelial A549 cell line was cultured in DMEM-F12 (1:1) with GlutaMAX (Gibco) at 37 °C in a 5% CO_2_ atmosphere. Every culture medium was supplemented with 10% heat-inactivated fetal bovine serum (FBS), 100 units/ml penicillin, and 100 µg/ml streptomycin mix (Gibco), 1 mM sodium pyruvate (Gibco), 10 mM HEPES (Gibco), and 5 × 10^–5^ mol/l 2-mercaptoethanol (Life Technologies).

For THP-1 differentiation into macrophages, cells were cultured in 24-well plates for 3 h with 300 ng/ml PMA at a density of 5 or 6 × 10^5^ cells per well, then cells were washed in PBS and cultured 3 days in complete RPMI 1640.

### Reagents

Nigericin, LPS from *E. coli* strain 0111:B4, and BFA were from Sigma-Aldrich. Phorbol 12-myristate 13-acetate (PMA), MSU, ATP, alum, glybenclamide, Ac-YVAD-cmk, MCC950 (kind gift from F. Niedergang) were from Invivogen. Primary polyclonal rabbit anti-FADD (2782), monoclonal rabbit IgG anti-NLRP3 clone D2P5E, monoclonal rabbit IgG anti-CASPASE-1 clone D7F20, polyclonal rabbit anti-CASPASE-4 (4450), monoclonal mouse anti-CASPASE-8 (1C12), and monoclonal rabbit IgG clone E1E3I anti-ASC antibodies were from Cell Signaling Technologies. Monoclonal mouse anti-α-Tubulin clone DM1A and monoclonal ECL horseradish peroxidase-conjugated-linked mouse anti-β-Actin clone AC-15 were from Sigma-Aldrich. Secondary antibodies used for immunoblot, ECL horseradish peroxidase-conjugated-linked donkey antibody (F(ab′)2 fragment) anti-rabbit IgG (NA9340V), and ECL horseradish peroxidase-conjugated-linked goat anti-mouse IgG1 (M32107), were from GE Healthcare and Caltag, respectively.

### Human samples

Experiments were approved by the local ethics review committees and written informed consent was obtained from all patients. The age and sex of the HD and patients are provided in Supplementary Table 2.

Human primary monocytes were isolated from heparinized peripheral blood or buffy coats of 9 HD from the Etablissement Français du Sang (EFS) and 10 RA patients fulfilling the American College of Rheumatology (ACR) classification for RA who have been treated at the Rheumatology Department of the Cochin Hospital (Paris, France). PBMCs-derived monocytes were isolated using a double gradient centrifugation according to the published protocol^56^ with Pancoll human density 1.077 g/ml (Pan-Biotech) and a 46% iso-osmotic Percoll solution (Percoll, density: 1.131 g/ml, GE Healthcare). Presence of monocytes was confirmed by labeling with monoclonal mouse IgG2a anti-CD14-QDot655 clone TüK4 (1:100 dilution; Invitrogen), monoclonal mouse IgG1 anti-CD16-APC-H7 clone 3G8 (1:40 dilution; BD Biosciences) and monoclonal mouse IgG1 anti-CD11c-Alexa Fluor 700 clone 3.9 (1:10 dilution; eBioscience) antibodies (kind gift from S. Isnard), and analysis on LSRII flow cytometer (BD Biosciences).

Serum samples were obtained for 534 RA suffering patients from the ESPOIR cohort^46^, a French national observational multi-centric cohort of patients having arthritis starting since more than 6 weeks and <6 months and not undergoing any treatment with synthetic or biologic disease-modifying antirheumatic drugs (DMARDs) at inclusion. One biological resources center (Joelle Benessiano, Paris-Bichat) was in charge of centralizing and managing the biologic data collection of the cohort. Serum samples from 14 gout-suffering patients were obtained from the Rheumatology Department at the Lariboisière Hospital (Paris, France). Serum samples from 33 HD were obtained from the EFS from 5 ml of whole blood centrifuged at 2000 × *g* for 15 min and immediately frozen and stored at −80 °C.

Synovial fluids were obtained from the knee of five OA and nine RA-suffering patients who have been treated at La Cavale Blanche Hospital (Brest, France). Synovial fluids from four gout-suffering patients were obtained from the Rheumatology Department at the Lariboisière Hospital and synovial fluids from eight additional OA-suffering patients were obtained from the Rheumatology Department of the Cochin Hospital. Synovial fluids were centrifuged at 1300 × *g* for 30 min and stored at −80 °C.

### Structural assessment

Patients with early RA included in the ESPOIR cohort undergo radiographic evaluation every 6 months during the first 2 years of follow-up. Radiographs of the hands and feet (antero-posterior views) were collected in the radiography coordinating center. X-rays obtained at baseline and at 1 and 2 years of follow-up were read in a standardized way. All sets of X-rays were read by a trained investigator blinded to clinical evaluation. Structural damage was assessed qualitatively by the presence of typical RA erosions according to their location and aspect and rated according to the van der Heijde-modified Sharp score (mSHS)^57^ on radiographs of both hands and wrists, and feet. Radiographic progression was defined as an increase in mSHS > 1, assessed between baseline and the end of years 1 and 2 of follow-up. RRP was defined as an increase in mSHS > 10 within the first 2 years of follow-up (i.e., 5 points per year).

### CRISPR/Cas9-mediated gene targeting

Guide RNAs were designed using http://crispr.mit.edu/ and were: 5′-GCTAATGATCGACTTCAATG-3′ (NLRP3), 5′-GCTGGAGAACCTGACCGCCG-3′ (ASC), 5′-GATTGACTCCGTTATTCCGAA-3′ (CASP1), and 5′-GGGATGAAGGAGCTACTTGA-3′ (CASP4). Guide RNAs were cloned into pSpCas9(BB)-2A-GFP (PX458), which was a gift from Feng Zhang, according to the published protocol^58^. Cas-9-mediated cleavage efficiency was evaluated using the GeneArt Genomic Cleavage Detection Kit (Life Technologies). THP-1 cells were then transfected with the plasmids using the Amaxa Nucleofector II device (Lonza) and the Human Monocyte Nucleofector Kit (Lonza) as described^59^. GFP-positive transfected cells were sorted and plated on 96-well plates at a density of 1 cell per well by flow cytometry using BD FACSARIA III (BD Biosciences) and cultivated for 14–21 days. Viable clonal cell lines were then cultivated in 24-well plates and half of the cells were lysed using QuickExtract DNA extraction solution (Epicentre) to perform an amplification of the region of interest from genomic DNA by PCR using AmpliTaq master Mix (Life Technologies). PCR primers used were: *NLRP3* forward, 5′-TGGGATTACAGGCGTGAGC-3′, and reverse, 5′-TCTCTCCTGTTGATCGCAGC-3′; *ASC* forward, 5′-AGACCAGAGTGGGAGGAAG-3′, and reverse, 5′-AGGAGGAACAGAAAGCGGAAG-3′; *CASP1* forward 5′-GATGTGAGAAATCCTTGTGC-3′, and reverse 5′-CAGAAGAGTGCCACTGA-3′; and CASP4 forward, 5′-TAAAGGAGAGAAACAACCGCAC-3′, and reverse, 5′-GATTTTCGGGGCTGCATCC-3′. PCR products were then sequenced by Sanger sequencing (Eurofins Genomics) using forward primers used for PCR. Sequences were analyzed using http://tide-calculator.nki.nl/ (ref. ^60^) to identify insertion/deletion mutations. Clonal cell lines with defined mutations were then assessed by western blotting and/or functional assay.

### Cell stimulation

THP-1, U-937, K-562, Jurkat, MOLT-4, and HBP-ALL cells were primed or not with LPS (100 ng/ml) overnight in RPMI 1640 with 2% FBS. Then cells were stimulated in cell culture insert with 0.4 µm pore size in 400 µl RPMI 1640 without FBS in 24-well plates that contained 100 µl of the same medium, at a density of 4 × 10^6^ cells per well. PMA-differentiated THP-1 cells were stimulated in RPMI 1640 with 0% or 2% FBS in 24-well plates at a density of 5 or 6 × 10^5^ cells per well. HeLa, HaCat, and A549 cells were seeded at a density of 4 × 10^5^ cells per well in 24-well plates that contained culture medium with 2% FBS. Isolated human primary PBMCs-derived monocytes were primed or not with LPS (200 ng/ml) overnight in RPMI 1640 with 5% FBS and stimulated in RPMI 1640 without FBS in 24-well plates at a density of 1 × 10^6^ cells per well. If not otherwise indicated, NLRP3 activation of all the cell lines was achieved with a final concentration of 20 µM of nigericin for 1 h, 500 µg/ml of MSU or alum for 6 h, with live *E. coli* strain O127:H6 (EPEC, E2348/69, kind gift from Julie Guignot) at a multiplicity of infection of 20 for 2.5 h followed by a 16 h incubation following addition of 100 units/ml penicillin and 100 µg/ml streptomycin to kill living bacteria. NLRP3 activation of primary PBMCs-derived monocytes was achieved with a final concentration of 5 µM of nigericin for 1 h or with 5 mM of ATP for 30 min. Inhibition of potassium efflux was achieved by addition in the media of 130 mM KCl or 50 µM of glybenclamide. Caspase-1 inhibition was achieved using 10 µM of Ac-YVAD-cmk. NLRP3 inflammasome inhibition was achieved using 1 or 5 µM of MCC950. Classical secretion inhibition was achieved using 5 µg/ml of BFA. Following cells stimulation, culture supernatants were collected either directly or under the insert when the cells were cultured in cell culture inserts. Directly collected supernatants were next filtrated on culture inserts with 0.4 µm pore size. All supernatants were then stored at −20 °C. Concurrently, cell death was evaluated using trypan blue exclusion, LDH activity assay kit (Sigma) or propidium iodide (Life Technologies) uptake according to the manufacturer recommendations. Propodium iodide-stained cells were analyzed using ACCURI C6 flow cytometer (BD Biosciences).

### ELISA

To detect soluble FADD, PBS-Tween 20 (PBS-T) was added to the biological samples to obtain a final concentration of 0.05% Tween 20 that were then heated for 5 min at 100 °C. For the ESPOIR cohort and the OA/RA synovial fluids (Fig. 5f), FADD levels were measured using previously described ELISA^34^. Otherwise, FADD levels were measured by homemade quantitative sandwich ELISA using monoclonal mouse anti-FADD antibody clone A66-2 (2.5 µg/ml in PBS; BD Biosciences) for coating overnight at 4 °C. PBS containing 2% bovine serum albumin (BSA) was used for blocking of non-specific-binding sites. Samples and recombinant human FADD protein (Sigma-Aldrich) that was used to perform standard range were incubated 2 h at room temperature (RT) followed by an incubation overnight at 4 °C. Polyclonal rabbit anti-FADD (1:250 in PBS-T 0.05% 2% BSA; Cell Signaling Technologies) was used for detection at 2 h RT followed by ECL horseradish peroxidase-conjugated-linked donkey antibody (F(ab′)2 fragment) anti-rabbit IgG (1:5,000 in PBS-T 0.05% 2% BSA; GE Healthcare) for revelation 45 min at RT. The colorimetric reaction was revealed by adding 3,3′,5,5′-tetramethyl-benzidine (TMB). Reaction was stopped with 2 N sulfuric acid and the plates were read at 450 nm with a EL800 microplate reader (BioTek Instruments). IL-1β levels were measured using human IL-1 beta ELISA Ready-SET-Go!^®^ kit (eBioscience) according to the manufacturer’s instructions. When cells were cultured through a trans-well membrane (400 nm pores), secreted FADD or IL-1β quantity was calculated reporting FADD concentration measured by ELISA to the total volume passed through the trans-well.

### Western blotting

Cells were washed in PBS, and total proteins were extracted using lysis buffer (10 mM Tris–HCl, 150 mM NaCl pH 7.8, 1% Nonidet P-40 (Sigma-Aldrich), containing a protease inhibitors cocktail (complete mini EDTA-free, Roche Diagnostics)), and sample concentrations were determined using Micro BCA Protein Assay (Thermo Fisher Scientific). Laemmli buffer was added to total proteins (40 µg) or to 40 µl of collected culture supernatant or to microvesicles isolated from 15 × 10^6^ cells and boiled for 5 min. Then samples were subjected to SDS–PAGE, transferred to polyvinylidene difluoride (PVDF) membrane (PerkinElmer Life Sciences). PVDF membrane was then blocked with Tris-buffered saline (TBS) containing 5% nonfat dry milk and then probed with specific primary polyclonal rabbit anti-FADD (1:1000 dilution, Cell Signaling Technologies), monoclonal rabbit IgG anti-NLRP3 clone D2P5E (1:1000 dilution), monoclonal rabbit IgG anti-CASPASE-1 clone D7F20 (1:1000 dilution), polyclonal rabbit anti-CASPASE-4 (1:500 dilution), monoclonal mouse anti-CASPASE-8 clone 1C12 (1:1000 dilution), monoclonal rabbit IgG clone E1E3I anti-ASC (1:1000 dilution), monoclonal mouse anti-α-Tubulin clone DM1A (1:350) or monoclonal ECL horseradish peroxidase-conjugated-linked mouse anti-β-Actin clone AC-15 (1:10,000 dilution) antibodies (all described in the reagent section) diluted in TBS Tween 20 (TTBS) 0.1% containing 5% nonfat dry milk or BSA overnight at 4 °C, according to the manufacturer’s recommendations. Secondary ECL horseradish peroxidase-conjugated-linked donkey antibody (F(ab′)2 fragment) anti-rabbit IgG or ECL horseradish peroxidase-conjugated-linked goat anti-mouse IgG1 antibodies were diluted 1:5000 in TTBS 0.1% containing 5% nonfat dry milk. Immunoreactivity was visualized using the enhanced chemiluminescence (ECL) technique (Amersham Biosciences) and images were acquired using a Fusion FX7 system imaging (Vilbert Lourmat).

### Isolation of EVs

THP-1 cells were cultured 1 h in the absence or presence of 20 µM nigericin in RPMI 1640 medium without FBS^61^. Following incubation, culture supernatants were harvested and EVs were isolated through differential centrifugation. Briefly, supernatants were successively centrifuged at 300 × *g* for 5 min to eliminate the cells, 2000 × *g* for 10 min to eliminate cell debris and apoptotic bodies, 10,000 × *g* for 30 min to isolate the microvesicles that were then washed once in 0.2 µm filtrated-PBS. Supernatants were then centrifuged at 100,000 × *g* for 90 min using L-70 ultracentrifuge with SW55 Ti rotor (Beckman) to separate the soluble proteins and the small vesicles, including exosomes, that were washed once in 0.2 µm filtrated-PBS.

### Liquid chromatography and tandem mass spectrometry (LC–MS/MS)

#### Sample preparation

10,000 × *g* microvesicle pellets fractions from mock-treated or nigericin-treated THP-1 cells were lysed and denatured in Tris 50 mM pH 8.5 and SDS 2% while disulfide bridges were reduced using TCEP 20 mM and subsequent free thiols groups were protected using chloroacetamide 50 mM for 5 min at 95 °C. Proteins (15 µg estimate) were trypsin-digested overnight using the filtered-aided sample preparation (FASP) method to collect peptides as previously described^62^. After desalting on C_18_ home-made Stage-tips, 100 ng of each peptide extract were analyzed.

#### LC–MS/MS

Peptides were concentrated, separated and analyzed with an Ultimate 3000 Rapid Separation liquid chromatographic system coupled to an Orbitrap Fusion mass spectrometer (both from Thermo Fisher Scientific). Briefly, peptides were loaded on a C_18_ reverse phase precolumn (3 μm particle size, 100 Å pore size, 75 μm inner diameter, 2 cm length, from Thermo Fischer Scientific) using loading solvent (1% Acetonitrile and 0.1% trifluoroacetic acid in milliQ H_2_O) for 3 min at 5 µl/min, then separated on an analytical C_18_ reverse phase column (2 µm particle size, 100 Å pore size, 75 µm internal diameter, 25 cm length) with a 90 min effective gradient from 99% A (0.1% formic acid in milliQ H_2_O) to 50% B (80% Acetonitrile, 0.085% formic acid in milliQ H_2_O) at 400 nl/min. The mass spectrometer acquired data throughout the LC elution process and operated in a data-dependent scheme with full MS scans acquired with the orbitrap, followed by HCD fragmentation and Ion trap fragment detection (top speed mode in 5 s) on the most abundant ions detected in the MS scan. Mass spectrometer settings were for full scan MS: AGC: 2.0E4, target resolution: 60,000, *m*/*z* range was 350–1500, maximum ion injection time: 60 ms; for HCD MS/MS: quadrupole filtering, normalized collision energy: 27. Ion trap rapid detection, intensity threshold: 1.0E4, isolation window: 1.6*m*/*z*, dynamic exclusion time: 30 s, AGC target: 2.0E4 and maximum injection time: 100 ms. The fragmentation was permitted for precursor with charge state of 2–7. The software used to generate.mgf peaklists files was Proteome discoverer 1.4 (Thermo Fisher Scientific).

#### Peptide identification

Peak lists were used to perform comparison of experimental MS/MS data with the *Homo sapiens* taxon of the Swiss-Prot database (February 2016, 20,273 sequences) using Mascot version 2.5.1 (http://www.matrixscience.com). The cleavage specificity was trypsin’s with maximum 1 missed cleavages. The precursor mass tolerance was set to 4 ppm and the MS/MS mass tolerance to 0.55 Da. Cystein carbamidomethylation was set as a complete modification while methionine oxidation was set as variable. With these settings, peptides identifications were considered as valid whenever their scores reached a minimum of 25, thus meeting the *p*-values criteria <0.01. Data output were filtered and compared using MyPROMS version 3.1 (ref. ^63^). Proteins identified with false discovery rate below 1% and at least two distinct peptides in one of the samples were considered. Data sets were analyzed with FunRich^64^ for comparison with the Vesiclepedia database^44^. We considered proteins that have been identified only in the two independent experiments performed.

### Electron microscopy processing for ultrastructure

Cell were fixed for 1 h with 3% glutaraldehyde. Samples were postfixed in osmium tetroxide, 1% in 0.1 M phosphate buffer, then dehydrated in 70%, 90%, and finally 100% ethanol. After 10 min in a mixture of 1.2 epoxy propane and epoxy resin, the tissue was embedded in gelatin capsules with freshly prepared epoxy resin and polymerized at 60 °C for 24 h. 80-nm sections were cut with an ultramicrotome (Reichert ultracut S), stained with uranyl acetate and Reynold’s lead citrate, and observed with a transmission electron microscope (JEOL 1011).

### Immunogold electron microscopy

THP-1 or microvesicles isolated as previously described were fixed in 1% glutaraldehyde in 0.1 M phosphate buffer (pH 7.4), then embedded in sucrose, and frozen in liquid nitrogen. Cryosections were made using an ultracryomicrotome (Reichert Ultracut S.), and ultrathin sections mounted on Formvar-coated nickel grids were prepared. Sections were incubated with PBS 15% glycine (15 min), PBS 15% glycine 0.1% BSA (5 min), and PBS 15% glycine 0.1% BSA 10% normal donkey serum (20 min), followed by anti-FADD antibody (2 µg/ml, 2 h, Calbiochem) diluted in PBS 15% glycine 0.1% BSA and 4% normal donkey serum. After extensive rinsing, sections were incubated with gold-labeled secondary antibody with a gold particle size of 10 nm (British Biocell International), washed again, stained with 2% uranyl acetate (10 min), and air-dried. Sections were examined using a JEOL 1011 transmission electron microscope. Alternatively, microvesicles isolated as previously described were directly incubated on formwar-coated grids for 2 min, then with uranyl acetate 4% for 2 min. Immunogold labeling and observation were performed as described above using anti-FADD antibody (Cell Signaling Technologies).

### Statistical tests

Statistical analyses were performed using non-parametric tests as the data did not fulfill the criteria of normal distribution and equal variance. Quantitative data analyses using the Mann–Whitney test were performed with RStudio. Statistical details for each experiment can be found in the corresponding figure legend. “*N*” corresponds to the number of individual donors/patients or the number of samples. Correlation analyses using the Spearman test were performed with the GraphPad Prism7 software. Odds ratios and 95% confidence intervals were calculated. *P* values < 0.05 were considered to indicate statistical significance. In figures, NS stands for not significant and asterisks stand for: *P < 0.05, **P < 0.01, ***P < 0.001.

## Supplementary information

Unmarked supplemental material

Supplemental Figure 1

Supplemental Figure 2

Supplemental Figure 3

## Data Availability

The mass spectrometry proteomics data reported in this paper have been deposited to the ProteomeXchange Consortium via the PRIDE^65^ partner repository with the dataset identifier PXD008168.
