# Supplementary material for: The classical NLRP3 inflammasome controls FADD unconventional secretion through microvesicle shedding
Source: Cell Death Dis. 2019 Feb 25;10(3):190. doi: 10.1038/s41419-019-1412-9 (PMC6389912; doi:10.1038/s41419-019-1412-9)
Supplement: Supplementary file 1 — Unmarked supplemental material [file 41419_2019_1412_MOESM1_ESM.docx]

Supplementary Information for

**The classical NLRP3 inflammasome controls FADD unconventional secretion through microvesicle shedding**

Sara Mouasni^1,2,3,4^, Virginie Gonzalez^1,2,3,4^, Alain Schmitt^2,3,4,5^, Evangeline Bennana^2,3,4,6^, François Guillonneau^2,3,4,6^, Sylvie Mistou^1,2,3,4^, Jérôme Avouac^1,2,3,4,7^, Hang Korng Ea^8,9,10^, Valérie Devauchelle^11^, Jacques-Eric Gottenberg^12^, Gilles Chiocchia^13,14^ & Léa Tourneur^*,1,2,3,4^

^1^Department of Infection, Immunity and Inflammation, Cochin Institute, 75014 Paris, France; ^2^Inserm, U1016, Paris, France ; ^3^Cnrs, UMR8104, Paris, France ; ^4^Paris Descartes University, Sorbonne Paris Cité, Paris, France ; ^5^Cochin Imaging: Electron microscopy facility, 75014 Paris, France; ^6^3P5 Proteomics facility, 75014 Paris, France; ^7^Rheumatology Department, AP-HP, Cochin Hospital, 75014 Paris, France; ^8^Rheumatology Department, Viggo-Petersen Center, AP-HP, Lariboisière Hospital, 75010 Paris, France; ^9^University School of Medicine, University Paris-Cité Paris-Diderot, Paris, France; ^10^INSERM UMR-S U1132, Paris, France; ^11^Rheumatology Department, CHU la Cavale Blanche, 29200 Brest, France ; ^12^Rheumatology Department, Strasbourg University Hospitals, 67000 Strasbourg, France; ^13^Inserm U1173, University of Versailles-Saint-Quentin, Saint-Quentin-En-Yvelines, France ; and ^14^UFR des Sciences de la Santé, Simone Veil, 78180 Montigny-Le-Bretonneux, France

^*^Corresponding author: L Tourneur, Institut Cochin, Département Infection, Immunité, Inflammation, INSERM U1016, CNRS UMRS 8104, Université Paris Descartes UMRS 1016, Bâtiment Gustave Roussy, 27 rue du faubourg Saint-Jacques 75014 Paris; Tel: +33 1 40516606 ; Fax: +33 1 40516601 ; Email: lea.remy-tourneur@parisdescartes.fr

**Running Title:** NLRP3 inflammasome induces FADD secretion

**Supplemental Fig. 1.** NLRP3 inflammasome activation induces IL-1β secretion from THP-1 human cell line. **a** Dose effect of nigericin on IL-1β protein secretion. ELISA quantification of IL-1β secreted by THP-1 monocytic cell line cultured for 1 h with (□) or without (■) different doses of nigericin (N=4). **b** Kinetics of IL-1β release in response to nigericin. ELISA quantification of IL-1β secreted by THP-1 cells cultured with (□) or without (■) 20 µM nigericin during indicated times (N=3 to 8, depending time point). **c** Dose effect of nigericin on THP-1 cellular viability. Cell death was evaluated by trypan blue exclusion following 1 h incubation of THP-1 monocytic cell line with (□) or without (■) different doses of nigericin (N=6). **d** Kinetics of THP-1 cell death in response to nigericin. Cell death was evaluated by trypan blue exclusion following incubation during indicated times of THP-1 cells cultured with (□) or without (■) 20 µM nigericin (N=3 to 8, depending time point). **e** ELISA quantification of IL-1β secreted by THP-1 cells cultured for 1 h with (+) or without (-) 20 µM nigericin (N=82). **f** Cell death evaluated by trypan blue exclusion from THP-1 monocytes following 1 h culture with (+) (N=89) or without (-) (N=95) 20 µM nigericin. **g** Correlation between THP-1 monocyte mortality (evaluated by trypan blue exclusion) and spontaneous (left panel, N=77) and nigericin-induced (right panel, N=89) IL-1β secretion levels (determined by ELISA). **h** Cell death evaluated by trypan blue exclusion (TBE) or propidium iodide staining and cytometry analysis (PI) from THP-1 monocytes following 1 h culture with (+) or without (-) 20 µM nigericin (N=3). **i** Effect of LPS priming on IL-1β secretion. ELISA quantification of IL-1β secreted by THP-1 cells not primed (-) or primed by 100 ng/ml LPS overnight (+) followed by stimulation 1 h with (+) or without (-) 20 µM nigericin (N=6). **j** IL-1β secretion by macrophages. ELISA quantification of IL-1β secreted by PMA-differentiated THP-1 cells cultured with (+) or without (-) nigericin (20 µM during 1 h, n=8), monosodium urate (MSU, 500 µg/ml during 6 h, N=5) or alum (500 µg/ml during 6 h, N=7). **k** Effect of diluents on FADD secretion by THP-1 cells. Med, medium; EtOH, ethanol. H20 is the diluent for ATP and alum; DMSO is the diluent for Glybenclamide (Fig. 2 and Supplemental Fig. 2); PBS is the diluent for MSU; ethanol is the diluent for Nigericin and Brefeldin A (Fig. 4 and Supplemental Fig. 3a). Each diluent was used at the concentration corresponding to the highest concentration used with drugs of interest. FADD concentrations were measured by ELISA after one hour of incubation. **l** Western blot analysis of CASPASE-4 (CASP4), NLRP3, ASC, CASPASE-1 (CASP1) and FADD proteins in wild type (WT) and *CASPASE-4*-KO (*CASP4* KO) THP-1 cell line; α-tubulin serves as a loading control. * indicates non-specific immunoreactive band. **m** ELISA quantification of IL-1β secreted by PBMCs-derived monocytes from healthy donors not primed (-) or primed by 200 ng/ml LPS overnight (+) followed by stimulation in absence (-) or presence (+) of nigericin (5 µM during 1 h, N=9) or ATP (5 mM during 30 min, N=7). **n** Cell death evaluated by LDH release from PBMCs-derived monocytes from healthy donors not primed (-) or primed by 200 ng/ml LPS overnight (+) followed by stimulation in absence (-) or presence (+) of nigericin (5 µM during 1 h, N=9) or ATP (5 mM during 30 min, N=7). Each symbol represents one donor and bar represents mean ± sem (**m, n**).

Cells were cultured through a trans-well membrane (400 nm pores) (**a-j, m, n**). IL-1β or FADD quantity was calculated reporting concentration measured by ELISA to the total volume passed through the trans-well (**a, b, e, i-k, m**). Results are expressed as mean ± sem (**a-f, i-k**). Mann-Whitney test was performed, * *P* < 0.05, ** *P* < 0.01, *** *P* < 0.001.

**Supplemental Fig. 2.** IL-1β secretion requires potassium efflux but not extracellular glucose. **a** ELISA quantification of IL-1β secreted by THP-1 cells cultured for 1 h with (+) or without (-) 20 µM nigericin in absence (-) or presence (+) of potassium efflux inhibitors (130 mM KCl, N=6 to 10; 50 µM glybenclamide, N=9 to 12). **b** ELISA quantification of IL-1β secreted by PBMCs-derived monocytes from healthy donors primed by LPS (200 ng/ml overnight) followed by 1 h stimulation with (+) or without (-) 5 µM nigericin in absence (-) or presence (+) of 50 µM glybenclamide (N=3 to 5). Each symbol represents one donor. **c** Cell death evaluated by LDH release from PBMCs-derived monocytes from healthy donors primed by LPS (200 ng/ml overnight) followed by 1 h stimulation with (+) or without (-) 5 µM nigericin in absence (-) or presence (+) of 50 µM glybenclamide (N=3 to 5). Each symbol represents one donor. **d** Generation of inflammasome-KO THP-1 cell clones. Western blot analysis of NLRP3, ASC, CASPASE-1 (CASP1) and FADD proteins in wild type (WT), *NLRP3*-KO, *ASC*-KO and *CASPASE-1*-KO (*CASP1* KO) THP-1 cell lines; α-tubulin serves as a loading control. Arrows indicate the three isoforms of the ASC protein. **e** ELISA quantification of IL-1β secreted by PBMCs-derived monocytes from healthy donors primed by LPS (200 ng/ml overnight) and pretreated for 1 h in absence (-) or in presence (+) of 1 or 5 µM of the NLRP3 inflammasome inhibitor MCC950, followed by 1 h stimulation in absence (-) or presence (+) of 5 µM nigericin; and cell death evaluated by LDH release from the same PBMCs-derived monocytes (N=2; each symbol represents one donor); **f** ELISA quantification of IL-1β and FADD secreted by THP-1 monocytes cultured for 1 h with (+) or without (-) 20 µM nigericin diluted in RPMI 1640 medium or in PBS buffer or in PBS with calcium and magnesium (N=6); and cell death evaluation by trypan blue exclusion from the same THP-1 cells. **g** Cell death evaluated by trypan blue exclusion from THP-1 cells cultured for 1 h with (+) or without (-) 20 µM nigericin diluted in RPMI 1640 medium or in PBS buffer in absence (-) or presence (+) of 2 mg/ml of glucose (N=6). RPMI, RPMI 1640 medium.

Cells were cultured through a trans-well membrane (400 nm pores) (**a-c, e-g**). IL-1β or FADD quantity was calculated reporting concentration measured by ELISA to the total volume passed through the trans-well (**a, b, e, f**). Results are expressed as mean ± sem (**a, f, g**). Mann-Whitney test was performed, * *P* < 0.05, ** *P* < 0.01, *** *P* < 0.001.

**Supplemental Fig. 3.** NLRP3 inflammasome activation triggers extracellular vesicles secretion. **a** ELISA quantification of IL-1β and FADD secreted by THP-1 monocytes cultured for 1 h with (+) or without (-) 20 µM nigericin in absence (-) or presence (+) of 10 µg/ml of the ER-Golgi transport inhibitor brefeldin A (N=8). **b, c** Extracellular vesicles from THP-1 cells cultured for 1 h with (+) or without (-) 20 µM nigericin were isolated by 10,000 x *g* (**b**) or 100,000 x *g* (**c**) centrifugation, then analyzed by FACS using MACS Quant system or by electron microscopy. Arrows indicate vesicles. MV, microvesicle; Exo, exosome. **d** Western blot quantification of FADD protein contained within THP-1 cells (Lysate) following 1 h culture with (+) or without (-) 20 µM nigericin, and within microvesicles (MV, isolated from supernatant from 15.10^6^ cells by 10,000 x *g* centrifugation) and soluble proteins (SP, obtained from supernatant from 15.10^6^ cells by 100,000 x *g* centrifugation) (N=3). β-actin expression is used for normalization. **e** Culture supernatants from THP-1 monocytic cell line cultured for 1 h with different doses of nigericin, as indicated, were treated with (■) or without (□) 0.05% Tween 20 to induce MV burst (N=4). Then ELISA quantification of FADD was performed. **f** ELISA quantification of FADD and IL-1β contained within extracellular vesicles (EV) and soluble proteins (SP) secreted by THP-1 monocytes following 1 h incubation with (+) or without (-) 20 µM nigericin (N=7). Culture supernatants were obtained by differential centrifugation as described in the materials and methods section. Results are expressed as mean ± sem (**e, f**). Mann-Whitney test was performed, * *P* < 0.05, ** *P* < 0.01, *** *P* < 0.001. NS, not significant.

**Supplemental Table 1.** Lack of peptide signal in the FADD protein sequence.

| **FADD protein specie** | **Signal Peptide Prediction** | | | | | | | | **Protein Secretion Prediction** | | |
| --- | --- | --- | --- | --- | --- | --- | --- | --- | --- | --- | --- |
|  | Phobius | Signal-3L | Signal-BLAST | SignalP | Predotar | Spoct-opus | Predi  Si | SecretomeP | Predi  Si | | SecretomeP |
| Chimpanzee  (*P. troglodytes*) | Yes | No | No | No | No | No | Yes^*^ | No | Yes | Yes / UPS | |
| Mouse  (*M. musculus*) | Yes | No | No | No | No | No | Yes^*^ | Yes | Yes | Yes | |
| Rat  (*R. norvegicus*) | Yes | No | No | No | No | No | No | Yes | No | Yes | |
| Dog  (*C. familiaris*) | Yes | No | No | No | No | No | No | No | No | Yes / UPS | |
| Bovine  (*B. taurus*) | Yes | No | No | No | No | No | Yes | No | Yes | Yes / UPS | |
| Sheep  (*O. aries*) | Yes | No | No | No | No | No | Yes | Yes | Yes | Yes | |
| Pig  (*S. scrofa*) | Yes | No | No | No | No | No | Yes | No | Yes | Yes / UPS | |
| Tasmanian devil  (*S. harrisii*) | No | No | No | No | No | No | No | No | No | Yes / UPS | |
| Chicken  (*G. gallus*) | Yes | No | No | No | No | No | Yes | - | Yes | - | |
| Xenopus  (*X. tropicalis*) | No | No | No | No | No | No | No | - | No | - | |
| Zebrafish  (*D. renio*) | No | No | No | No | No | No | No | - | No | - | |
| Mediterranean mussel (*M. galloprovincialis*) | No | No | No | No | No | No | No | - | No | - | |
| Fruit fly  (*D. melanogaster*) | No | No | No | No | No | No | No | - | No | - | |

^*^Score >0.50: sequence very likely contains a signal peptide; chimpanzee FADD score: 0.52; mouse FADD score: 0.504.

- : not determined.

**Supplemental Table 2.** Donor and patient characteristics

|  |  | BDM | Serum | Synovial fluid |
| --- | --- | --- | --- | --- |
|  | n= | 11 | 33 | - |
| HD | Females (%) | 72.7 | 39.4 | - |
|  | Age^*^ (y) | 31.0 ± 2.6 (22-42)^†^ | 39.7 ± 2.3 (21-64) | - |
|  | n= | - | - | 13 |
| OA | Females (%) | - | - | 69.2 |
|  | Age (y) | - | - | 67.7 ± 2.1 (55-82) |
|  | n= | 10 | 534 | 8 |
| RA | Females (%) | 60.0 | 74.9 | 75.0 |
|  | Age (y) | 57.3 ± 2.2 (48-68) | 48.0 ± 0.5 (17-73) | 61.3 ± 3.4 (51-76)^‡^ |
|  | n= | - | 14 | 4 |
| Gout | Females (%) | - | 7.4 | 0.0 |
|  | Age (y) | - | 47.4 ± 3.5 (27-68) | 50.3 ± 9.1 (27-68) |

^*^Results are expressed as mean ± SEM (min-max)

^†^Data available for 7/11 donors

^‡^Data available for 6/8 patients

- : Irrelevant

Abbreviations: BDM, Blood-Derived Monocytes; HD, Healthy Donors; OA, Osteoarthritis suffering patients; RA, Rheumatoid Arthritis suffering patients; y, years.
