# Supplementary figures and images for: The classical NLRP3 inflammasome controls FADD unconventional secretion through microvesicle shedding
Source: Cell Death Dis. 2019 Feb 25;10(3):190. doi: 10.1038/s41419-019-1412-9 (PMC6389912; doi:10.1038/s41419-019-1412-9)

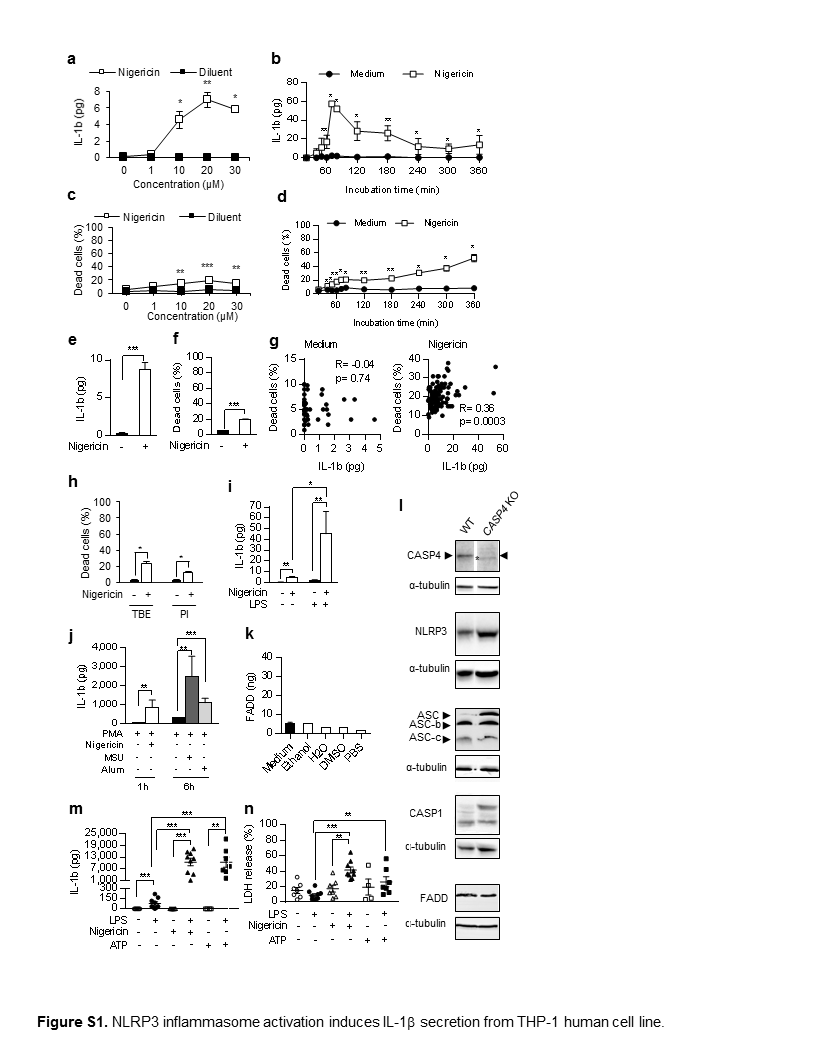

Supplement: Supplementary file 2 — Supplemental Figure 1 [file 41419_2019_1412_MOESM2_ESM.tif]

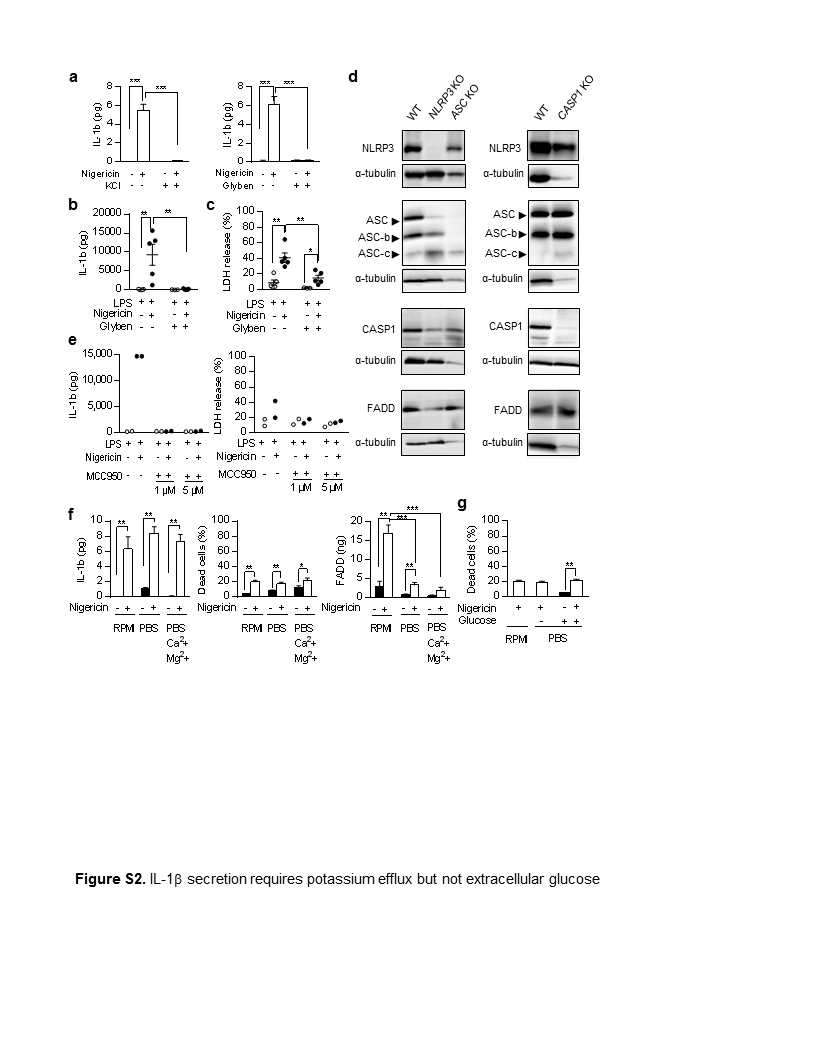

Supplement: Supplementary file 3 — Supplemental Figure 2 [file 41419_2019_1412_MOESM3_ESM.tif]

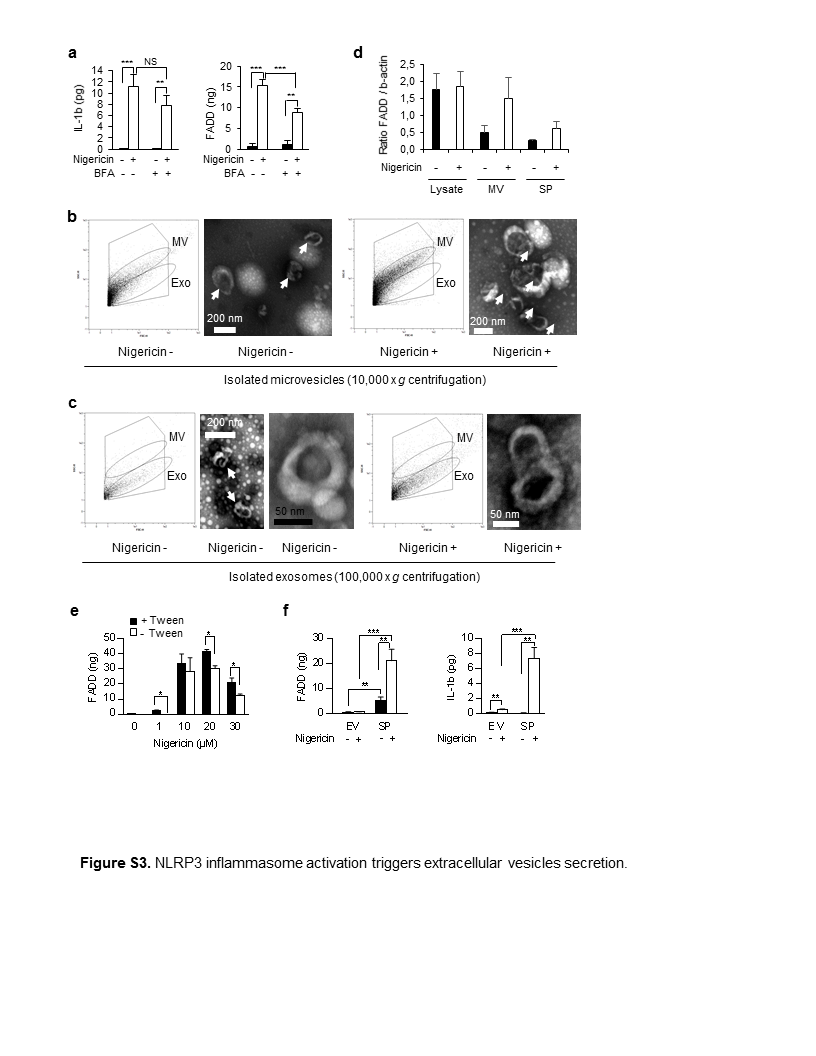

Supplement: Supplementary file 4 — Supplemental Figure 3 [file 41419_2019_1412_MOESM4_ESM.tif]
